# Investigating Conservation of the Cell-Cycle-Regulated Transcriptional Program in the Fungal Pathogen, *Cryptococcus neoformans*

**DOI:** 10.1371/journal.pgen.1006453

**Published:** 2016-12-05

**Authors:** Christina M. Kelliher, Adam R. Leman, Crystal S. Sierra, Steven B. Haase

**Affiliations:** Department of Biology, Duke University, Durham, North Carolina, United States of America; University College Dublin, IRELAND

## Abstract

The pathogenic yeast *Cryptococcus neoformans* causes fungal meningitis in immune-compromised patients. Cell proliferation in the budding yeast form is required for *C*. *neoformans* to infect human hosts, and virulence factors such as capsule formation and melanin production are affected by cell-cycle perturbation. Thus, understanding cell-cycle regulation is critical for a full understanding of virulence factors for disease. Our group and others have demonstrated that a large fraction of genes in *Saccharomyces cerevisiae* is expressed periodically during the cell cycle, and that proper regulation of this transcriptional program is important for proper cell division. Despite the evolutionary divergence of the two budding yeasts, we found that a similar percentage of all genes (~20%) is periodically expressed during the cell cycle in both yeasts. However, the temporal ordering of periodic expression has diverged for some orthologous cell-cycle genes, especially those related to bud emergence and bud growth. Genes regulating DNA replication and mitosis exhibited a conserved ordering in both yeasts, suggesting that essential cell-cycle processes are conserved in periodicity and in timing of expression (i.e. duplication before division). In *S*. *cerevisiae* cells, we have proposed that an interconnected network of periodic transcription factors (TFs) controls the bulk of the cell-cycle transcriptional program. We found that temporal ordering of orthologous network TFs was not always maintained; however, the TF network topology at cell-cycle commitment appears to be conserved in *C*. *neoformans*. During the *C*. *neoformans* cell cycle, DNA replication genes, mitosis genes, and 40 genes involved in virulence are periodically expressed. Future work toward understanding the gene regulatory network that controls cell-cycle genes is critical for developing novel antifungals to inhibit pathogen proliferation.

## Author Summary

The opportunistic fungal pathogen *Cryptococcus neoformans* infects immune-compromised humans and causes fungal meningitis by proliferating in the central nervous system. The cell cycle has not been studied at the whole transcriptome level in *C*. *neoformans*. Here, we present the expression dynamics of all genes from a synchronous population of *C*. *neoformans* cells over multiple cell cycles. Our study shows that almost 20% of all *C*. *neoformans* genes are periodically expressed during the cell cycle. We also compare the program of cell-cycle-regulated transcription in *C*. *neoformans* to the well-studied but evolutionary distant yeast, *Saccharomyces cerevisiae*. We find that many orthologous cell-cycle genes are highly conserved in expression pattern (e.g. DNA replication and mitosis genes), while others, notably budding genes, have diverged in expression ordering. We also identify 40 virulence genes from previous studies that are periodically expressed during the *C*. *neoformans* cell cycle in rich media. Our findings indicate that a conserved set of fungal transcription factors (TFs) controls the expression of conserved cell-cycle genes, while other periodic transcripts are likely controlled by species-specific TFs.

## Introduction

About 500 million years of evolution separate the fungal phyla Ascomycota and Basidiomycota [1,2]. The cell cycle is an essential biological process driving cell division of these distantly related yeasts, and therefore may be under strong selective pressure for conservation. Both *Saccharomyces cerevisiae* (Ascomycota) and *Cryptococcus neoformans* (Basidiomycota) can grow and divide asymmetrically in a budding yeast form. *C*. *neoformans* is a causative agent of deadly fungal meningitis, primarily in immune-compromised patients [3,4]. Many groups studying *C*. *neoformans* focus on virulence factors for human infection, such as the yeast’s polysaccharide capsule, melanin production, Titan cell formation, and others [5–9]. We propose that the function of cell-cycle regulators, which are essential for proliferation in the host, merit further investigation as virulence factors. Furthermore, there is evidence that virulence pathways are perturbed when cell-cycle progression is slowed, which suggests direct connections between cell-cycle regulators and virulence pathways [10,11].

The cell cycle is the process by which a cell duplicates its contents and faithfully divides into two genetically identical cells. In eukaryotes, a biochemical oscillator drives sequential cell-cycle events, where the cyclin-dependent kinase (CDK) and its variety of cyclin binding partners initiate events by phosphorylation, followed by destruction of kinase activity in mitosis by the anaphase-promoting complex (APC). Another common feature of the eukaryotic cell cycle is a temporally regulated program of transcription, which has been demonstrated in *S*. *cerevisiae*, *Schizosaccharomyces pombe*, *Arabidopsis thaliana*, mouse fibroblasts, and human tissue culture cells [12–22]. These programs of periodic genes include cyclin mRNAs, DNA replication factors, APC activators, and other cellular components that are utilized at specific times during the cell cycle. Our group and others have proposed that this “just-in-time transcription” mechanism is an important aspect of energy-efficient and faithful cell divisions [23,24]. In *S*. *cerevisiae*, an interconnected network of periodic transcription factors (TFs) is capable of driving the periodic program of cell-cycle gene expression [15,25–27]. Aspects of this yeast TF network are conserved in human cells; for example, G2/M genes are activated by a periodic forkhead domain-containing TF in both eukaryotes [22,28]. The topology of cell-cycle entry is also functionally conserved, where a repressor (*S*.*c*. *WHI5*, *H*.*s*. RB1) is removed by G1 cyclin/CDK phosphorylation to activate a G1/S transcription factor complex (*S*.*c*. SBF/MBF, *H*.*s*. E2F-TFDP1) [29]. However, the genes involved in cell-cycle entry are not conserved at the sequence level between fungi and mammals [30], suggesting that the fungal pathway could be targeted with drugs without affecting mammalian host cells.

Sequence-specific DNA-binding TFs have been identified in *C*. *neoformans* and phenotypically profiled by single gene knockouts [6,31,32]. This TF deletion collection was profiled over many virulence factor-inducing conditions to discover pathways that regulate disease and drug response genes [32]. Serial activation of TFs during capsule production has also been studied to elucidate the order in which TFs control virulence gene products [31]. However, the cell cycle has not been investigated in synchronous populations of cells to date. Although the phenotypes of some single mutant cell-cycle TFs have been examined from asynchronous populations, these studies offer limited understanding of temporal aspects of gene expression during the cell cycle.

Here we investigate transcriptional dynamics of the pathogenic yeast *C*. *neoformans* using cells synchronized in the cell cycle. We compare our findings to the cell-cycle transcriptional program in *S*. *cerevisiae*. We find that a similar percentage of all genes (~20%) are periodically transcribed during the cell cycle, and we present a comprehensive periodicity analysis for all expressed genes in both yeasts. We show that S-phase gene orthologs are highly conserved and temporally precede M-phase gene orthologs in both yeasts. Additionally, we find that many TFs in the cell-cycle entry pathway are conserved in sequence homology, periodicity, and timing of expression in *C*. *neoformans*, while others, notably genes involved in budding, are not. We also identify 40 virulence genes that appear to be cell-cycle-regulated, along with nearly 100 orthologous fungal genes that are periodic in the same cell-cycle phase. Taken together, these cell-cycle genes represent candidates for further study and for novel antifungal drug development.

## Results

### Cell-cycle synchronization and determination of periodic gene expression

**Fig 1:**
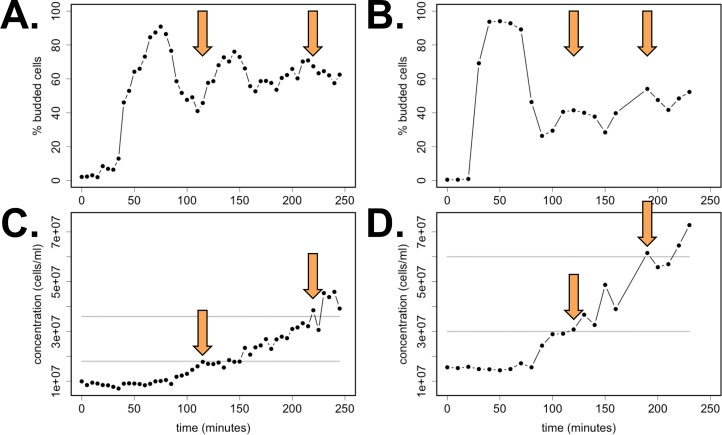
Population synchrony for *S*. *cerevisiae* and *C*. *neoformans* over > 2 cell cycles. *S*. *cerevisiae* cells were grown in 2% YEPD media, synchronized by alpha-factor mating pheromone, and released into YEPD (**A**) *C*. *neoformans* cells were grown in 2% YEPD rich media; small daughter cells were isolated by centrifugal elutriation and released into YEPD (**B**). Population synchrony was estimated by counting at least 200 cells per time point for the presence or absence of a bud, and doubling time was also monitored (**C-D**). Orange arrows indicate the time points where each population passed a complete doubling in cell concentration from the previous cycle (gray lines).

Identifying approaches for synchronizing populations of *C*. *neoformans* has been challenging. We succeeded in synchronizing by centrifugal elutriation, a method that has been very successful for *S*. *cerevisiae* cells [15,27,33]. For *C*. *neoformans*, we isolated early G1 daughter cells by centrifugal elutriation and released the population into rich media (YEPD) at 30°C to monitor cell-cycle progression, as described previously [34]. This size-gradient synchrony procedure is conceptually similar to the *C*. *neoformans* synchrony procedure presented by Raclavsky and colleagues [35]. For *S*. *cerevisiae*, we isolated G1 cells by alpha-factor mating pheromone treatment [36]. We utilized this synchrony technique to isolate larger *S*. *cerevisiae* cells and to offset some loss of synchrony over time due to asymmetric cell divisions. A functional mating pheromone peptide for *C*. *neoformans* has been described but is difficult to synthesize in suitable quantities [37]. After release from synchronization, bud formation and population doubling were counted for at least 200 cells over time (Fig 1). The period of bud emergence was about 75 minutes in both budding yeasts grown in rich media, although the synchrony of bud emergence after the first bud in *C*. *neoformans* appeared to be less robust (Fig 1A and 1B). Each yeast population completed more than two population doublings over the course of the experiments.

Total RNA was extracted from yeast cells at each time point (every 5 minutes for *S*. *cerevisiae*, or every 10 minutes for *C*. *neoformans*) and multiplexed for stranded RNA-Sequencing. Between 87–92% of reads mapped uniquely to the respective yeast genomes (S1 File). To identify periodic genes, we applied periodicity algorithms to the time series gene expression datasets. Four algorithms were used to determine periodicity rankings for all genes in each yeast: de Lichtenberg, JTK-CYCLE, Lomb-Scargle, and persistent homology [38–42]. Since each algorithm favors slightly different periodic curve shapes [43], we summed the periodicity rankings from each algorithm and ranked all yeast genes by cumulative scores for *S*. *cerevisiae* and for *C*. *neoformans* (S1 Table and S2 Table, respectively). By visual inspection, the top 1600 ranked genes in both yeasts appeared periodically transcribed during the cell cycle (S1 Fig). There was no clear “threshold” between periodic and non-periodic genes during the cell cycle—rather, we observed a distribution of gene expression shapes and signatures over time (S1 Fig). Previous work on the *S*. *cerevisiae* cell cycle has reported lists ranging from 400–1200 periodic genes. To validate our RNA-Sequencing time series dataset for the *S*. *cerevisiae* cell cycle, we compared the top-ranked 1600 periodic genes to previously published cell-cycle gene lists and found a 57–89% range of overlap with previous periodic gene lists (S2 Fig) [12–15,33,41,44,45].

**Fig 2:**
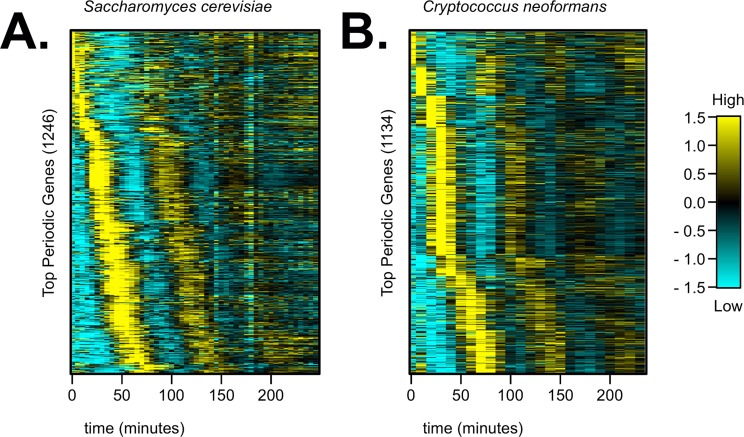
About 20% of all *S*. *cerevisiae* and *C*. *neoformans* genes are periodically expressed during the cell cycle. Four periodicity-ranking algorithms were run on the time series gene expression datasets at a period of 75 minutes (see S1 File). The top-ranked periodic genes (1–1600) were then filtered by the Lomb-Scargle algorithm to identify (**A**) 1246 periodic genes in *S*. *cerevisiae* and (**B**) 1134 periodic genes in *C*. *neoformans*. Genes in each periodic gene list were ordered along the y-axis by peak time of expression in the respective yeast dataset. As expected, the second and third cell cycles showed expression level damping due to asymmetric cell divisions in both budding yeasts. Transcript levels are depicted as a z-score change relative to mean expression for each gene, where values represent the number of standard deviations away from the mean. Each row represents transcript levels of a unique gene across the time series. Each column represents a time point in minutes.

Three filters were applied to each budding yeast dataset to estimate and compare the number of periodic genes (S1 File). First, we pruned noisy, low-expression genes from each dataset, leaving 5913 expressed genes in *S*. *cerevisiae* (S1 Table) and 6182 expressed genes in *C*. *neoformans* (S2 Table). Next, we took the top 1600 expressed genes from the cumulative ranking of the four periodicity algorithms described above. Finally, we applied a score cutoff to each list of top 1600 genes using the Lomb-Scargle algorithm (see S1 File) [39,40,43]. We estimated that there are 1246 periodic genes in *S*. *cerevisiae* (~21% expressed genes) and 1134 periodic genes in *C*. *neoformans* (~18% expressed genes) (Fig 2). We also provided multiple criteria for evaluating the cell-cycle expression patterns of individual genes in each yeast (S1 Table, S2 Table, S1 Fig).

Cellular processes that contribute to virulence are a major focus of work in the *C*. *neoformans* field. We took advantage of the partial *C*. *neoformans* deletion collection and genetic screens for virulence factors [6] and searched for periodic virulence genes. We found that 40 genes (about 16% of the virulence genes characterized by the Madhani group and many previous studies) were periodically expressed in *C*. *neoformans* during the cell cycle (S3 Table). These virulence genes are periodic during normal cycles in rich media, which suggests that some virulence processes are directly cell-cycle-regulated. For example, budding and cell wall synthesis are coupled to cell-cycle progression in *S*. *cerevisiae*. A subset of 14 periodic virulence genes in *C*. *neoformans* had capsule and/or cell wall phenotypes reported in previous studies (S3 Table). We then asked if the 40 periodic virulence genes might be co-regulated during the *C*. *neoformans* cell cycle (S3 Fig). Over half of the periodic virulence genes clustered together and peaked in a similar cell-cycle phase (20–30 minutes into cycle 1). 11 of the 14 capsule / cell wall genes were contained in this cluster (S3 Fig, S3 Table).

**Fig 3:**
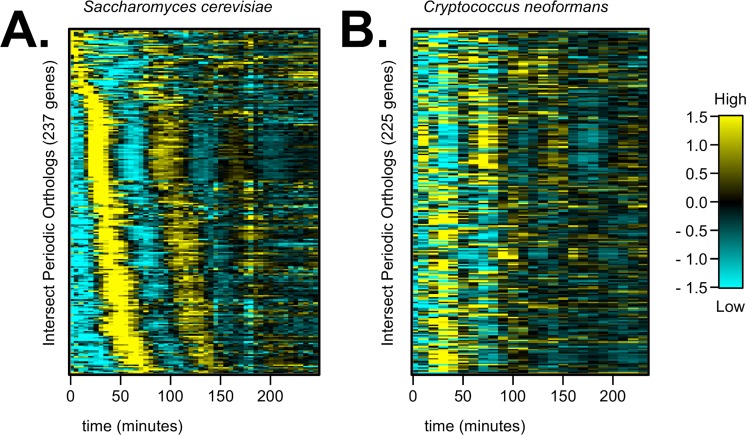
Periodic, orthologous genes between *S*. *cerevisiae* and *C*. *neoformans* are differentially ordered during the cell cycle. In *S*. *cerevisiae*, 753 genes out of the 1246 periodic genes had at least one ortholog in *C*. *neoformans* (60.4%). In *C*. *neoformans*, 593 genes out of the 1134 periodic genes had at least one ortholog in *S*. *cerevisiae* (52.3%). The intersection of these two gene lists contained 237 unique *S*. *cerevisiae* (**A**) and 225 unique *C*. *neoformans* (**B**) gene orthologs that were periodic in both budding yeasts. *C*. *neoformans* orthologs were plotted in the same relative order as their ortholog in *S*. *cerevisiae* (**B**), and we observed that many periodic genes have diverged in temporal ordering between the two yeasts. Transcript levels are depicted as a z-score change relative to mean expression for each gene, where values represent the number of standard deviations away from the mean. Orthologous periodic gene pairs are in the same relative order for (**A-B**) (for exact ordering of gene pairs and multiple-mapping orthologs, see S5 Table). Each column represents a time point in minutes.

Next, we wanted to ask if periodicity and temporal ordering of orthologous genes is evolutionarily conserved between the two budding yeasts. We compiled the largest list to date of putative sequence orthologs between *C*. *neoformans* and *S*. *cerevisiae* from the literature, databases, and additional BLAST searches (S1 File, S4 Table) [32,46–48]. About half of the periodic genes from each yeast (Fig 2) had at least one sequence ortholog in the other species. However, there were only about 230 pairs of orthologous genes that were labeled periodic in both yeasts. Those pairs of periodic orthologs have diverged in temporal ordering between *C*. *neoformans* and *S*. *cerevisiae* (Fig 3, S5 Table). These results indicated that the programs of periodic gene expression, and possibly the regulatory pathway, have diverged to some degree between the two budding yeasts. This altered temporal ordering between *S*. *cerevisiae* and *C*. *neoformans* periodic orthologous genes was likely not due to the experimental synchrony procedure. We obtained transcriptome data from two previous studies on *S*. *cerevisiae* cell-cycle-regulated transcription (which applied a different cell-cycle synchrony procedure, used different lab strains of *S*. *cerevisiae*, and/or measured gene expression on different platforms), and our list of periodic *S*. *cerevisiae* genes maintained temporal ordering during the cell cycle in all three datasets (S4 Fig).

Cell-cycle regulated gene expression has also been investigated in a species of pathogenic Ascomycota, *Candida albicans* [49]. To ask about common periodic gene expression in an evolutionarily intermediate budding yeast species, we further identified putative periodic orthologous genes shared between *S*. *cerevisiae*, *C*. *neoformans*, and *C*. *albicans*. A core set of almost 100 orthologs appeared to have both conserved periodicity and temporal ordering between all three budding yeasts (S5 Fig, S5 Table). This fungal gene set was enriched for functions in mitotic cell cycle and cell-cycle processes, which suggested that core cell-cycle regulators are under strong selection for conservation at the sequence level and by timing of periodic gene expression.

### Conservation of known cell-cycle regulators

We reasoned that some cell-cycle events must be invariable in temporal ordering between fungi (S5 Fig). DNA replication (S-phase) should be highly conserved across organisms because duplication of genetic material is essential for successful division. Segregation of genomic content during mitosis (M-phase) is also essential for division, and duplication must precede division. Using annotations for *S*. *cerevisiae* [50] we identified lists of genes known to be involved in regulating events in various cell-cycle phases including bud formation and growth [51,52], DNA replication [53,54], and spindle formation, mitosis, and mitotic exit [55–58]. We filtered the resulting gene lists by periodicity in *S*. *cerevisiae* (Fig 2A, S6 Table). We then identified orthologous genes in *C*. *neoformans* without enforcing a periodicity filter.

We have previously shown that expression timing of canonical cell-cycle orthologs in *S*. *cerevisiae* and *S*. *pombe* can vary—some gene pairs shared expression patterns while others diverged [59]. To temporally align orthologous gene plots between *S*. *cerevisiae* and *C*. *neoformans*, we used the algorithmic approach described previously with *S*. *cerevisiae* and *S*. *pombe* time series transcriptome data [59]. The first, most synchronous cycle of budding data from each yeast was fit using the CLOCCS algorithm (Fig 1, S6 Fig) [59,60]. Time points in minutes were then transformed into cell-cycle lifeline points to visualize the data (see S1 File).

**Fig 4:**
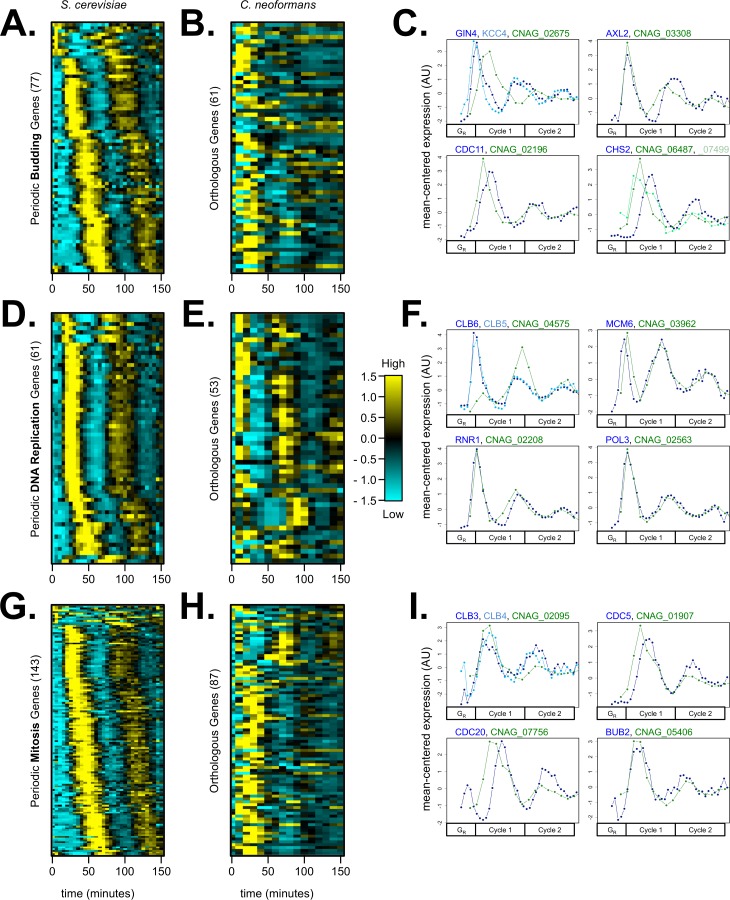
DNA replication, spindle assembly, and mitosis genes are highly conserved in temporal ordering during the fungal cell cycles, while budding orthologs vary in their temporal expression pattern in *C*. *neoformans*. *S*. *cerevisiae* genes annotated as bud assembly and growth genes were identified and filtered by periodicity (77 genes) (**A**). Many budding genes had an ortholog in *C*. *neoformans* (61 genes, 79.2%), and some orthologs were labeled periodic (20 genes, 32.8%) (**B**). Genes annotated as DNA replication genes were identified and filtered by periodicity (61 genes) (**D**). Almost all DNA replication genes had an ortholog in *C*. *neoformans* (53 genes, 86.9%), and over half of the orthologs were labeled periodic (28 genes, 52.8%) (**E**). Genes annotated as mitotic and spindle assembly genes were also identified and filtered by periodicity (143 genes) (**G**). Over half of the M-phase genes had an ortholog in *C*. *neoformans* (87 genes, 60.8%), and many orthologs were called periodic (53 genes, 60.9%) (**H**). Transcript levels are depicted as a z-score change relative to mean expression for each gene, where values represent the number of standard deviations away from the mean. Orthologous periodic gene pairs are in the same order for (**A-B**, **D-E**, or **G-H**) (for exact ordering of gene pairs and multiple-mapping orthologs, see S6 Table). Each column represents a time point in minutes. Canonical budding (**C**), DNA replication (**F**), and mitotic (**I**) gene orthologs are plotted to compare transcript dynamics between *S*. *cerevisiae* (blue) and *C*. *neoformans* (green). Global alignment E-values for ortholog pairs can be found in S4 Table. Line plots for orthologs are shown on a mean-normalized scale (same linear scaling method as heatmaps) (**C**, **F**, and **I**). This mean-normalization was used because *C*. *neoformans* genes have higher fold-change expression levels than *S*. *cerevisiae* genes (S1 Fig). Orthologous genes are plotted on a common cell-cycle timeline in CLOCCS lifeline points as described (see S1 File). In both yeasts, S-phase genes generally precede M-phase genes in temporal order (**D-F**, **G-I**).

As observed previously, *S*. *cerevisiae* genes that regulate budding, S-phase, and mitosis were largely transcribed periodically in the proper phases (Fig 4A, 4D and 4G) [12–15]. Cell-cycle gene expression peak time patterns were examined to quantitatively compare cell-cycle phases (S7 Fig). Bud assembly and growth genes peaked throughout the cell-cycle transcription program, and the temporal ordering of these genes repeated across cell cycles (Fig 4A, S7A and S7B Fig). Similarly, spindle assembly and mitosis genes peaked in the mid-to-late phases of the transcription program (Fig 4G). DNA replication genes peaked in a defined window in the middle phase of the transcription program (Fig 4D). We observed analogous expression patterns for *C*. *neoformans* orthologs associated with S-phase and mitosis (Fig 4E and 4H), but orthologs associated with budding appeared to be expressed with less restriction to a discrete cell-cycle phase or strict temporal order (S7 Fig). This budding gene pattern can be observed qualitatively where the unrestricted expression timing creates a more “speckled” appearance in the *C*. *neoformans* heatmap (Fig 4B) and differentially timed gene expression peaks (Fig 4C).

We hypothesize that bud emergence and bud growth are not as tightly coordinated with cell-cycle progression in *C*. *neoformans* cells. Unlike *S*. *cerevisiae* where bud emergence occurs primarily at the G1/S transition, *C*. *neoformans* bud emergence can occur in a broad interval from G1 to G2 phases [61,62]. The difference in budding transcript behaviors between *S*. *cerevisiae* and *C*. *neoformans* orthologs could therefore reflect the difference in the cell biology of bud emergence and growth (Fig 4A and 4B). Only about 33% of the orthologous budding gene pairs were periodically expressed in *C*. *neoformans*, compared to 53% DNA replication and 61% mitosis orthologs (Fig 4B, 4E and 4H). Furthermore, budding orthologs that were periodic in both *C*. *neoformans* and *S*. *cerevisiae* showed some divergence in expression timing (Fig 4C). We also observed that bud emergence of *C*. *neoformans* cells during the time series appeared less synchronous in second and third cycles than *S*. *cerevisiae* cells (Fig 1A and 1B). Bud emergence in *C*. *neoformans* could be controlled by both stress pathways and TF inputs because the first budding cycle is highly synchronous after elutriation synchrony, which causes a transient stress response in released cells (Fig 1B). However, our data do not rule out a model where some budding genes in *C*. *neoformans* are controlled post-transcriptionally by localization, phosphorylation, or other periodic mechanisms. It is also possible that budding orthologs are more difficult to identify than other cell-cycle genes due to sequence divergence or that novel budding genes have evolved in the *C*. *neoformans* lineage.

### Partial conservation of the transcription factor (TF) network control module

We have previously shown that a network of periodically expressed TFs is capable of driving the program of periodic genes during the *S*. *cerevisiae* cell cycle [15,27]. We hypothesized that a network of periodic TFs could also function in *C*. *neoformans* to drive a similar fraction of cell-cycle genes. Thus, the temporal re-ordering of part of the *C*. *neoformans* gene expression program (Fig 3) could be explained by two models: evolutionary re-wiring of shared network TFs with *S*. *cerevisiae* or novel TF network components arising in *C*. *neoformans* to drive cell-cycle genes. First, we asked if network TFs were conserved from *S*. *cerevisiae* to *C*. *neoformans*. Indeed, a majority of network TFs and key cell-cycle regulators have putative orthology between the two yeasts (Table 1) [30]. As observed for other cell-cycle genes (Fig 4), orthologs of some network TFs were expressed in the same phase in both yeasts, while others were expressed at different times (Table 1).

**Table 1:** TF network components in *S*. *cerevisiae* and sequence orthologs in *C*. *neoformans* have generally diverged in expression timing. Putative orthologous gene pairs were identified, if any (S4 Table) [30]. The peak time (minutes) and time to half-peak expression (minutes) was identified for the first cell cycle in each yeast. Peak times were similar for some pairs (e.g. *SWI4*, CNAG_07464), but many pairs have diverged in ordering (e.g. *FHL1*, CNAG_05934, and CNAG_05535). The protein global alignment score is also shown for each putative ortholog pair (S4 Table). Some reported ortholog pairs did not have a significant global alignment score (i.e. E-value > 10), which was likely due to similar local sequence matches (e.g. homologous protein domains) and divergent regions elsewhere in the proteins (see S1 File).

*S*. *cerevisiae* gene ID	Peak time (minutes)	Half peak time (min)	*C*. *neoformans* gene ID	Peak time (minutes)	Half peak time (min)	Global E-Value
CDH1	5	0	CNAG_03191	30	20	9.80E-101
SWI4	20	15	CNAG_07464	10	0	>10
YOX1	20	15	CNAG_03229	70	10	>10
CLN1/2	25,25,5	15,15,0	CNAG_06092	10	0	0.78; 2.90E-02
HCM1	25	15	CNAG_03116	30	20	>10
STB1	25	15	NA			
CDC28	30	20	CNAG_01664	20	10	0
CLB3/4	35,45	25,30	CNAG_02095	30	20	1.2E-88; 4.40E-79
NDD1	40	25	NA			
FHL1	45	25	CNAG_05934	0	0	>10
FKH1	45	30	CNAG_05861	0	0	0.25
SWI6	45	25	CNAG_01438	10	0	1.50E-14
MBP1	45	30	CNAG_07464	10	0	6.80E-41
FHL1	45	25	CNAG_05535	50	40	>10
YHP1	45	15	CNAG_03229	70	10	>10
NRM1	45	20	NA			
CLB1/2/5/6	50,65,25,20	40,40,15,15	CNAG_04575	80	60	5.8E-72; 9.30E-91; >10
WHI5	50	25	CNAG_05591	10	0	>10
FKH2	55	30	CNAG_02566	10	0	2.90E-04
ACE2	60	40	NA			
SWI5	60	45	NA			
CDC20	65	50	CNAG_07756	30	20	2.40E-90
MCM1	75	60	CNAG_07924	30	20	>10

**Fig 5:**
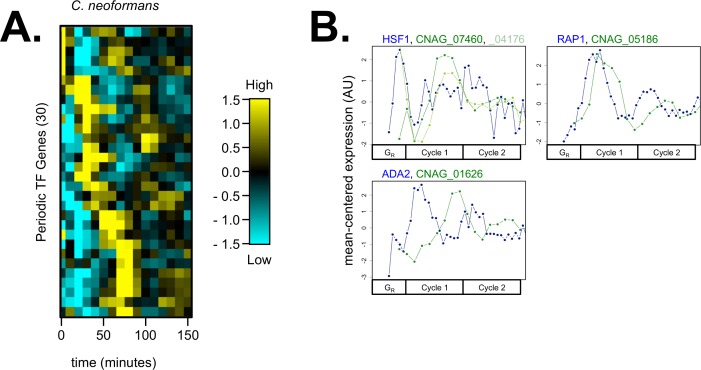
Novel periodic TFs in *C*. *neoformans* could regulate periodic gene expression. A gene list of *C*. *neoformans* TFs was obtained (180 genes) [32] and filtered by periodicity (36 genes, 20.0%). 6/36 periodic TFs were putative orthologs to previously known TFs in the *S*. *cerevisiae* cell-cycle network (Table 1). The remaining 30 novel periodic TFs are shown (**A**). Transcript levels are depicted as a z-score change relative to mean expression for each gene, where values represent the number of standard deviations away from the mean. Each column represents a time point in minutes. 18/30 periodic TFs have no documented ortholog in *S*. *cerevisiae*. 12/30 periodic TFs do have a putative ortholog in *S*. *cerevisiae*, but that gene is not currently known to participate in the *S*. *cerevisiae* cell-cycle network (S7 Table). Three examples of these ortholog pairs are shown between periodic *C*. *neoformans* TFs and their putative *S*. *cerevisiae* ortholog (**B**). Line plots for orthologs are shown on a mean-normalized scale (z-score of fpkm units, same linear scaling method as heatmaps) (**B**). This mean-normalization was used because *C*. *neoformans* genes have higher fold-change expression levels than *S*. *cerevisiae* genes (S1 Fig). Orthologous genes are plotted on a common cell-cycle timeline in CLOCCS lifeline points as described (see S1 File).

Second, we asked if there were any novel periodic TFs in *C*. *neoformans* (i.e. TFs with no predicted ortholog in *S*. *cerevisiae*, or TFs with an ortholog in *S*. *cerevisiae* that is not known to function in the TF network). We constructed a list of periodic *C*. *neoformans* TFs by filtering a previously annotated transcription factor list [32] with our list of periodic genes (Fig 5, S7 Table). Indeed, 30 novel TF genes were periodic during the *C*. *neoformans* cell cycle (Fig 5A). Taken together, results from Table 1 and Fig 5 suggested that both network TF re-wiring and novel periodic TFs in *C*. *neoformans* could explain the differential ordering of periodic genes during the cell cycle (Fig 3).

**Fig 6:**
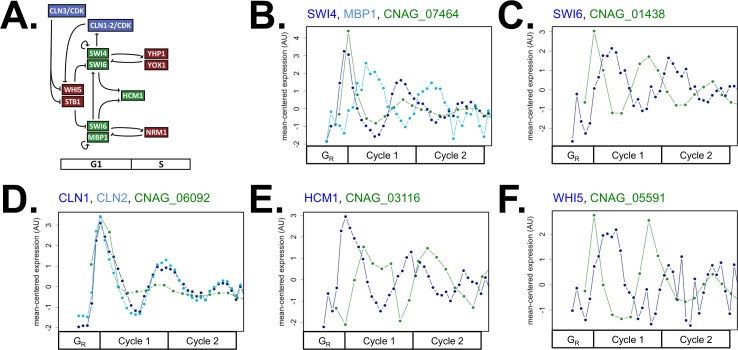
Evidence for conservation of the TF network topology at G1/S in *C*. *neoformans*. At cell-cycle entry in *S*. *cerevisiae*, the repressors Whi5 and Stb1 are removed from the SBF/MBF complexes by G1 cyclin/CDK phosphorylation. The heterodimeric TF complexes SBF (Swi4, Swi6) and MBF (Mbp1, Swi6) can then activate ~200 periodic genes at the G1/S border. SBF/MBF activate the downstream transcriptional activator Hcm1 to continue the temporal activation of S-phase genes. The transcriptional repressors Yox1, Yhp1, and Nrm1 then repress SBF/MBF (**A**). Ortholog pairs are shown for SBF/MBF (CNAG_07464 or MBS1) (**B**), *SWI6* (CNAG_01438 or MBS2) (**C**), G1 cyclins (CNAG_06092) (**D**), *HCM1* (CNAG_03116) (**E**), and *WHI5* (CNAG_05591) (**F**). Line plots for orthologs are shown on a mean-normalized scale (z-score of fpkm units, same linear scaling method as heatmaps) (**B-F**). This mean-normalization was used because *C*. *neoformans* genes have higher fold-change expression levels than *S*. *cerevisiae* genes (S1 Fig). Orthologous genes are plotted on a common cell-cycle timeline in CLOCCS lifeline points as described (see S1 File).

Putative S-phase regulators in *C*. *neoformans* exhibited transcript behaviors that were very similar in periodicity and in ordering to their *S*. *cerevisiae* orthologs (Fig 4D–4F). Thus, we predicted that the network motifs and TFs controlling the transcription of periodic S-phase genes could be conserved. Orthologous genes in the G1/S topology were largely conserved in periodic expression dynamics at cell-cycle entry (Fig 6). The expression timing of some genes had shifted earlier in the *C*. *neoformans* cell cycle (Fig 6C and 6F, Table 1), but this result does not refute the hypothesis that these genes are activated and functional at G1/S phase. Therefore, the network topology of cell-cycle entry appeared largely conserved in *C*. *neoformans* both by sequence and by gene expression dynamics. The prediction of this model is that a common G1/S transcriptional network drives a common set of S-phase periodic genes. To test this model, we examined promoter sequences from TF network genes in *S*. *cerevisiae* and *C*. *neoformans*, as well as the promoters of 38 periodic DNA replication ortholog pairs, and did an unbiased search for enriched TF binding sequences. The core motif “ACGCGT” for SBF/MBF transcription factors [63–65] was identified in both *S*. *cerevisiae* and *C*. *neoformans* promoters. The motif was not enriched in randomly selected periodic gene promoters, suggesting that SBF/MBF is functionally conserved in *C*. *neoformans* to drive TF network oscillations and DNA replication gene expression (S8 Fig).

## Discussion

Here, we present the first RNA-Sequencing dataset of transcription dynamics during the cell cycle of *C*. *neoformans*. Despite evolutionary distance between Basidiomycota and Ascomycota, *S*. *cerevisiae* and its extensive genome annotation provided an excellent analytical benchmark to compare to cell-cycle transcription in *C*. *neoformans*. RNA-Sequencing has been shown to be more quantitative than microarray technology for lowly- and highly-expressed genes using asynchronous *S*. *cerevisiae* cells due to microarray background fluorescence and saturation of fluorescence, respectively [66].

We demonstrate that 20% or more of all genes in the budding yeast genomes are periodically transcribed during the cell cycle. A ranking of periodicity for transcript dynamics in *C*. *neoformans* is provided (S2 Table). For the sake of comparison, we have presented gene sets of 1100–1200 periodic genes with the highest relative periodicity scores as “cell-cycle-regulated”; however, there is a continuum of periodic gene expression dynamics during the cell cycle in both yeasts (S1 Fig). The four periodicity algorithms applied here yielded a range of periodicity scores with no clear distinction between “periodic” and “non-periodic” gene sets (S1 and S2 Tables). These results suggest that yeast mRNAs fluctuate in expression with various degrees of cell-cycle periodicity. We propose that the top 20% periodic genes presented in this study are directly regulated by periodic cell-cycle TFs in *C*. *neoformans* and in *S*. *cerevisiae*. We also posit that some of the remaining 80% genes are weakly cell-cycle regulated. For example, some genes could be subject to complex regulation with one regulatory input from a cell-cycle periodic TF and another input from a constitutively expressed TF.

We raise two important questions about the yeast periodic gene expression programs: is periodic expression of a core set(s) of genes required for the fungal cell cycle, and how are periodic gene dynamics controlled in each yeast?

In both yeasts, periodic transcription is a high dimensional cell-cycle phenotype because transcriptional state reflects the phase-specific biology of the cell cycle over repeated cycles (Fig 2 and Fig 4). In other words, G1-, S-, and M-phase genes follow a defined temporal ordering pattern. *S*. *cerevisiae* cells synchronized by different methods and/or grown in different conditions display similar ordering of periodic cell-cycle genes, despite different cell-cycle period lengths (S4 Fig). Here, we examined the transcriptome of cycling *C*. *neoformans* cells at 30°C. Other groups have shown that *C*. *neoformans* cells spend more time in G1 phase at 24°C [67]. We predict that future studies examining cell-cycle transcription of *C*. *neoformans* cells grown in different conditions (i.e. non-rich media or 37°C infection temperature) would continue to display a similar temporal ordering of cell-cycle genes. These findings provide more evidence that “just-in-time transcription” is a conserved feature of eukaryotic cell cycles [23].

We show that some orthologous periodic genes have diverged in temporal ordering during the cell cycles of *S*. *cerevisiae* and *C*. *neoformans* over evolutionary time (Fig 3). We specifically investigated genes that play a role in bud emergence and bud growth, and we find that many budding gene orthologs are not controlled in a defined temporal order during the *C*. *neoformans* cell cycle (Figs 1A, 1B, 4A and 4B). On the other hand, DNA replication and mitosis genes do appear to be conserved by sequence homology, periodic expression, and temporal ordering (Fig 4D–4I). Lastly, we find that a set of about 100 orthologous genes is both periodic and expressed in proper cell-cycle phase in the budding yeasts *S*. *cerevisiae*, *C*. *neoformans*, and *C*. *albicans* (S5 Fig) [49]. These findings suggest that there may be a conserved set of fungal cell-cycle-control genes, which represent novel therapeutic targets for fungal infections.

We posit that a network of periodic transcription factors (TFs) could control the periodic gene expression program in *C*. *neoformans*, which has been shown in *S*. *cerevisiae* and suggested in human cells [15,22,25,27]. Many orthologous genes to *S*. *cerevisiae* TF network components have diverged in expression timing in *C*. *neoformans* cells (Table 1). However, we show that the G1/S network topology is likely conserved between *S*. *cerevisiae* and *C*. *neoformans* because orthologous genes display similar expression dynamics (Fig 6). Furthermore, we find that the promoters of G1/S TF network orthologs and promoters of periodic DNA replication orthologs are enriched for an “ACGCGT” sequence motif, which matches the SBF/MBF binding site consensus in *S*. *cerevisiae* (S8 Fig) [63–65]. Therefore, we propose that the G1/S transcriptional motif—where a co-repressor is removed by G1 cyclin/CDK phosphorylation and a TF activator complex is de-repressed—is also conserved in *C*. *neoformans* (Fig 6B–6D and 6G) [29,30]. Downstream of the G1/S activator complex, the *C*. *neoformans* TF network may also contain a common forkhead domain S-phase activator and homeobox domain G1/S repressor (Fig 6E, Table 1) [14,68,69]. This partially conserved TF network model in *C*. *neoformans* explains the common G1/S topology, on-time DNA replication gene transcription, as well as differential expression of budding and other cell-cycle genes by divergent parts of the TF network.

The regulation of periodic transcription and the function of a putative TF network warrant further investigation as virulence factors of fungal meningitis caused by *C*. *neoformans*. It has been previously shown that fluconazole drug treatment can affect cell ploidy in *C*. *neoformans* [70]. More recently, polyploid Titan cells were shown to produce haploid and aneuploid daughter cells during *C*. *neoformans* infection [71]. Therefore, future work on proper regulation of DNA replication and the contribution of periodic gene products could greatly benefit our understanding of genome stability in *C*. *neoformans*. The *C*. *neoformans* TF deletion collection was recently phenotyped, and the potential of targeted TF therapies was discussed [32,72]. We have added to the *C*. *neoformans* genotype/phenotype map by documenting the functional outputs of cell-cycle TFs over synchronized cell cycles. We also propose that a conserved G1/S topology of cell-cycle TFs may initiate the cell-cycle transcription network in *C*. *neoformans*. It is possible that a multi-drug combination targeting cell-cycle regulators and previously characterized virulence pathways could yield more successful antifungal therapies [72]. For example, a combination therapy could target TFs at the conserved G1/S topology to slow cell-cycle entry and also target fungal cell wall or capsule growth. In the circadian rhythm field, it has been shown that drugs targeting Clock Controlled Genes are most potent when administered at the time of the target gene’s peak expression [73].

Interestingly, deletion of the known SBF/MBF ortholog, Mbs1 (CNAG_07464), is viable in *C*. *neoformans* [32,74]. These genetic results do not match *S*. *cerevisiae*, where *swi4 mbp1* double mutants are inviable [75]. In fact, deletion of the single known G1 cyclin ortholog, CNAG_06092, is also viable in *C*. *neoformans* [10]. Mbs1 and the G1 cyclin are likely important for cell-cycle progression in *C*. *neoformans* because mutant phenotypes are highly defective in capsule formation in G1 phase, melanin production, and response to Hydroxyurea treatment during S phase [10,11,32,74]. However, the genetics are inconsistent with findings in *S*. *cerevisiae* and warrant further investigation to characterize the G1/S TF network topology of *C*. *neoformans*. It is possible that uncharacterized, redundant genes exist in the *C*. *neoformans* G1/S network motif.

We find that 40 candidate virulence genes are periodically expressed during the *C*. *neoformans* cell cycle (S3 Table, S3 Fig). An important direction for future work is to identify the mechanistic links between cell-cycle regulators and virulence pathways. 14 periodic virulence genes have annotated phenotypes in capsule formation and/or cell wall secretion. Fungal cells must secrete new cell wall and capsule during growth, and the direct links between cell cycle and these virulence factors in *C*. *neoformans* warrants further study because the cell wall and capsule are not present in host cells. The ultimate goal of this work is to identify the regulatory mechanism of periodic gene expression in *C*. *neoformans* and to find optimal drug targets and combination therapies for disrupting the fungal cell cycle.

## Materials and Methods

### Yeast strains, cultures, and synchronization

The wild-type *Saccharomyces cerevisiae* strain is a derivative of BF264-15D MATa *bar1* [76,77]. The wild-type *Cryptococcus neoformans var*. *grubii* serotype A strain is a derivative of H99F [47]. Yeast cultures were grown in standard YEP medium (1% yeast extract, 2% peptone, 0.012% adenine, 0.006% uracil supplemented with 2% dextrose sugar). For centrifugal elutriation, cultures were grown in YEP-dextrose (YEPD) medium at 30°C overnight. Elutriated early G1 cells were then resuspended in fresh YEPD medium at 30°C for time series experiments. For α-factor arrest, cultures were grown in YEPD medium at 30°C and incubated with 30 ng/ml α-factor for about 110 minutes. Synchronized cultures were then resuspended in fresh YEPD medium at 30°C. Aliquots were taken at each time point and subsequently assayed by RNA-Sequencing.

### RNA isolation and RNA-sequencing analyses

Total RNA was isolated by acid phenol extraction as described previously [34]. Samples were submitted to the Duke Sequencing Facility (https://www.genome.duke.edu/cores-and-services/sequencing-and-genomic-technologies) for stranded library preparation and sequencing. mRNA was amplified and barcoded (Illumina TruSeq Stranded mRNA Library Preparation Kit for *S*. *cerevisiae* and KAPA Stranded mRNA-Seq Library Preparation Kit for *C*. *neoformans*) and reads were sequenced in accordance with standard Illumina HiSeq protocols. For *S*. *cerevisiae*, libraries of 50 base-pair single-end reads were prepared, and 10 samples were multiplexed and sequenced together in each single lane. For *C*. *neoformans*, libraries of 125 base-pair paired-end reads were prepared (due to larger and more complex yeast transcriptome with introns), and 12 samples were multiplexed and sequenced together in each single lane. Raw FASTQ files were aligned to the respective yeast genomes using STAR [78]. Aligned reads were assembled into transcripts, quantified, and normalized using Cufflinks2 [79]. Samples from each yeast time series were normalized together using the CuffNorm feature. The normalized output FPKM gene expression levels were used in the analyses presented. A detailed description of each analysis pipeline is presented in the S1 File.

### Accession numbers

RNA-Sequencing gene expression data from this manuscript have been submitted to the NCBI Gene Expression Omnibus (GEO; https://www.ncbi.nlm.nih.gov/geo/) under accession number GSE80474.

## Supporting Information

S1 File Supporting Information Methods. This file contains further details on the RNA-Seq data analysis pipeline, periodic gene ranking algorithms, alignment of the two time series experiments using CLOCCS, and documentation of sequence orthologs.
(DOCX)

S1 Table Ranking of periodic genes from the *S*. *cerevisiae* cell cycle. In the first column, genes are denoted by transcriptome GTF file gene IDs (typically gene common names). Scores or p-values and ranks from the 4 algorithms—persistent homology (PH), Lomb-Scargle (LS), JTK-CYCLE (JTK), and de Lichtenberg (DL)—are shown (columns 8–15). In the fourth column, a cumulative periodicity rank was calculated by adding the ranks from each algorithm (i.e. low cumulative ranks indicate optimal periodicity rankings by all algorithms). Mean expression for each gene is shown in the fifth column (fpkm units). The absolute amplitude was calculated in the sixth column by finding (max_expr_−min_expr_) (fpkm units). The fold-change was calculated in the seventh column by finding (max_expr_ / min_expr_) (fpkm units). Noisy genes were then pruned from the final ranking if more than half of the time series contained fpkm values less than 2 (1213 genes were marked “NA” in red). The second column, Normalized Periodicity Ranking, contains the remaining 5913 genes ranked by cumulative periodicity score with noisy genes removed. This column was used to determine the top 1600 periodic genes shown in S1 Fig. To further refine the *S*. *cerevisiae* periodic gene list, we re-ran the Lomb-Scargle algorithm to match the *C*. *neoformans* experimental time points (see S1 File). P-values from this LS run are provided in column 16. An LS cutoff was made, and genes passing the cutoff are highlighted in green in column 3. The final list of 1246 periodic genes (Fig 2A) was determined by 1) non-noisy genes, 2) genes in the top 1600 cumulative ranking, and 3) genes passing the LS cutoff. Column 17 contains the y-axis index for the 1246 periodic genes shown in Fig 2A.
(XLSX)

S2 Table Ranking of periodic genes from the *C*. *neoformans* cell cycle. In the first column, genes are denoted by transcriptome GTF file gene IDs (H99 accession standard names). Scores or p-values and ranks are shown from the 4 algorithms: PH, LS, JTK, and DL (columns 8–15). In the fourth column, a cumulative periodicity rank was calculated by adding the ranks from each algorithm (i.e. low cumulative ranks indicate optimal periodicity rankings by all algorithms). Mean expression for each gene is shown in the fifth column (fpkm units). The absolute amplitude was calculated in the sixth column by finding (max_expr_−min_expr_) (fpkm units). The fold-change was calculated in the seventh column by finding (max_expr_ / min_expr_) (fpkm units). Noisy genes were then pruned from the final ranking if more than half of the time series contained fpkm values less than 2 (780 genes were marked “NA” in red). The second column, Normalized Periodicity Ranking, contains the remaining 6182 genes ranked by cumulative periodicity score with noisy genes removed. This column was used to determine the top 1600 periodic genes shown in S1 Fig. To further refine the *C*. *neoformans* periodic gene list, we applied an LS p-value cutoff. Genes passing the cutoff are highlighted in green in column 3. The final list of 1134 periodic genes (Fig 2B) was determined by 1) non-noisy genes, 2) genes in the top 1600 cumulative ranking, and 3) genes passing the LS cutoff. Column 16 contains the y-axis index for the 1134 periodic genes shown in Fig 2B.
(XLSX)

S3 Table 40 genes associated with virulence phenotypes from previous studies are called periodic during the *C*. *neoformans* cell cycle. The Madhani group documented virulence genes from previous work and performed genetic screens for virulence factors from a partial *C*. *neoformans* deletion collection [6]. Their list of virulence genes and corresponding literature reference(s) was compiled (from Table 1, Table 2, S1 Table, and S2 Table [6]), and H99 accession IDs were assigned. 37 genes in red font were either identified through a modified FungiDB search or the gene ID could not be found [46]. Of the 257 genes assigned to a standard name, 40 are in the periodic gene list for *C*. *neoformans*. Columns 4 and 5 show literature references for each gene (with corresponding PMID) and key words for the virulence factor(s) reported in the respective study.
(XLSX)

S4 Table Documentation of 4572 pairs of sequence orthologs between *C*. *neoformans* and *S*. *cerevisiae*. Orthologous pairs (columns 1–2) were derived from FungiDB, literature supplemental materials, or manual BLAST searches (column 5) [32,46–48]. Duplicate mappings exist in both yeasts (i.e. 3405 unique *C*. *neoformans* genes and 3437 unique *S*. *cerevisiae* genes generate 4572 unique pairs). *S*. *cerevisiae* genes are also labeled with their standard gene ID (column 3) and any paralogs from the whole genome duplication (column 4, see S1 File for further details). Protein sequences from each fungal gene were obtained from FungiDB, and global alignments among all possible pairs were tested using the FASTA program [80]. The scores for each putative ortholog pair were extracted. Some pairs did not score significantly (E-value < 10) in global protein sequence alignment (marked with “NA”s). See the S1 File section “Documentation of sequence orthologs between *S*. *cerevisiae* and *C*. *neoformans*” for complete details.
(XLSX)

S5 Table Top periodic gene orthologs in both *S*. *cerevisiae* and *C*. *neoformans*, a subset of which are also periodic in *C*. *albicans*. To ask if orthologous pairs of genes are periodically expressed in both yeasts, we identified the intersection of genes in the periodic gene lists of both *S*. *cerevisiae* and *C*. *neoformans* (Fig 2). The overlapping orthologous gene pairs in Fig 3 represent ~19% of the top periodic genes shown in Fig 2 (237 unique *S*. *cerevisiae* and 225 unique *C*. *neoformans* genes, Excel Tab 1). For each ortholog pair (columns 1, 4), the periodicity rank from the respective yeast dataset is shown (columns 3, 6). Gene ordering by peak time of expression from the Fig 3 heatmaps is also shown (columns 2, 5). A subset of about 100 orthologous genes is also periodic during the *C*. *albicans* cell cycle (S5 Fig, Tab 2) [49]. For each ortholog pairing (columns 1, 3, 5), gene ordering by peak time of expression from the S5 Fig heatmaps is shown (columns 2, 4, 6)
(XLSX)

S6 Table Conservation of budding, S-phase, and M-phase genes. *S*. *cerevisiae* genes involved in bud formation and growth (154, Excel Tab 1, [50–52]), DNA replication (103, Excel Tab 2, [50,53,54]), and spindle formation, mitosis, and mitotic exit (258, Excel Tab 3, [50,55–58]) were identified and filtered by periodicity (columns 1, 3, 4). The *S*. *cerevisiae* periodic cell-cycle gene lists (77 budding, 61 DNA replication, 143 mitosis) were then queried for *C*. *neoformans* orthologs in budding (61), S-phase (53), and M-phase (87) genes, along with respective periodicity ranks (columns 5, 7, 8). Gene ordering by peak time of expression from Fig 4 is also shown (columns 2, 6).
(XLSX)

S7 Table Identification of novel periodic TFs in *C*. *neoformans*. A list of 178 *C*. *neoformans* TFs was taken from Jung and colleagues (column 1) [32], and 3 TFs were added manually (WHI5/CNAG_05591, FKH2/CNAG_02566, SWI4/CNAG_07464). Periodicity ranks are shown (columns 3, 4). The 74 *S*. *cerevisiae* orthologs and periodicity rankings are also shown (columns 5–7). Cells highlighted in green represent known cell-cycle network TFs in *S*. *cerevisiae*. Gene ordering by peak time of expression from Fig 5 is also shown (column 2).
(XLSX)

S1 Fig In both *Saccharomyces cerevisiae* and *Cryptococcus neoformans*, genes decay in periodicity as their ranking decreases. Four periodicity algorithms were run on both time series gene expression datasets at a period of 75 minutes. The top-ranked 1600 genes of *S*. *cerevisiae* (A-B) and *C*. *neoformans* (E-F) appear periodically expressed during the cell cycle. The next groups of ranked genes—1601–2400 (C, G) and 2401–3200 (D, H)—decay in periodic shape. However, there is no clear cutoff between “periodic” and “non-periodic” genes in either dataset. Transcript levels are depicted as a z-score change relative to mean expression for each gene. Each row represents a ranked periodic gene (see S1 and S2 Tables), and genes are ordered along the y-axis by peak expression during the cell cycle. Each column represents a time point in minutes. We also compared the distributions of amplitudes between *S*. *cerevisiae* (blue) and *C*. *neoformans* (green) ranked periodic genes (I-L). We examined two amplitude metrics—the absolute amplitude (max–min, top) and the fold-change amplitude (max / min, bottom). To compare the amplitude distributions, raw values were log2-normalized to make them normally distributed (I-L), and the following tests were conducted in R: wilcox.test, ks.test, var.test, and t.test. Distributions are statistically different for all fold-change histograms (I-L, bottom), where *C*. *neoformans* genes have higher mean fold-change values than *S*. *cerevisiae* genes. Distributions are statistically different for half of the absolute amplitude histograms (I, K, top), where *S*. *cerevisiae* genes have higher mean amplitude values than ranked *C*. *neoformans* genes.
(TIF)

S2 Fig Comparison of *Saccharomyces cerevisiae* wild-type periodic gene lists from nine studies. Periodic gene lists from each publication were derived as follows. The top 1600 genes from this study were converted to SGD standard names and 17 dubious ORFs were removed (1583 genes). The 856 microarray probe IDs from Bristow et al. Additional File 3 were converted to unique standard names (including duplicate probe ID mappings) to generate 881 genes (572 genes intersect with this study) [33]. The 479 genes from Eser et al. Addendum Table S6 were converted to standard names (425 intersect this study) [45]. The 598 genes from Granovskaia et al. Supplement Table 5 were converted to standard names, and 9 dubious ORFs were removed to generate 589 genes (487 intersect this study) [44]. The 1275 probe IDs from Orlando et al. Supplement Table 1 were converted to unique standard names to generate 1357 genes (777 intersect this study) [15]. The 991 genes from Pramila et al. with PBM5 rankings of 1000 or less were taken from Orlando et al., and 52 dubious ORFs were removed to generate 939 genes (618 intersect this study) [14]. The top 800 genes were taken from de Lichtenberg et al. (http://www.cbs.dtu.dk/cellcycle/yeast_benchmark/benchmark.php), and 47 dubious ORFs were removed to generate 753 genes (522 intersect this study) [41]. The 421 genes from Cho et al. were also taken from the de Lichtenberg et al. webpage, and 22 dubious ORFs were removed to generate 399 genes (326 intersect this study) [13]. The 800 genes from Spellman et al. were taken directly from the Supplement (http://genome-www.stanford.edu/cellcycle/data/rawdata/CellCycle95.xls), and 59 dubious ORFs were removed to generate 741 genes (540 intersect this study) [12]. Percent overlaps between each periodic gene list were calculated by dividing the number of intersecting genes by the total number of genes in the smaller list. Percent overlap is presented as a heatmap, and gene lists are ordered by date of publication.
(TIF)

S3 Fig 40 periodic virulence genes in *C*. *neoformans* cluster into two major cell-cycle phases. 40 periodic genes associated with virulence phenotypes from previous work (S3 Table) were clustered by an affinity propagation algorithm, as described in [15]. The 24 genes in Cluster A peak in an early-to-mid cell-cycle phase. The 16 genes in Cluster B are expressed approximately anti-phase to the Cluster A periodic genes. 11/14 periodic virulence genes associated with capsule and cell wall synthesis in *C*. *neoformans* belong to Cluster A (see S3 Table).
(TIF)

S4 Fig Periodic genes in *S*. *cerevisiae* share temporal ordering across a variety of synchrony procedures, experimental conditions, and gene expression measurement technologies. Microarray data was obtained from two different studies that profiled gene expression dynamics from wild-type yeast upon release from elutriation synchrony: Spellman 1998 [12] and Orlando 2008 [15]. Spellman and colleagues cultured the lab strain DBY7286 in YEP 2% ethanol at 25°C, elutriated, and released early G1 cells at 25°C. Orlando and colleagues cultured the lab strain 15D in YEP 2% galactose at 30°C, elutriated, and released early G1 cells into YEP 2% dextrose + 1 M Sorbitol at 30°C. In this study, cells were cultured in YEP 2% dextrose, arrested using alpha-factor, and G1 cells were released into YEP 2% dextrose at 30°C. 1214 out of 1246 periodic genes from this study (Fig 2A) were successfully mapped back to microarray probe IDs from the Affymetrix Yeast 2.0 array (Orlando) and to spots on custom-printed Cy3-Cy5 arrays (Spellman). In each heatmap, the 1214 genes were ordered in the exact same order along the y-axis by peak time of expression in the dataset from this study. For this study (A) and Orlando et al data (B), transcript levels are depicted as a z-score change relative to mean expression for each gene, where values represent the number of standard deviations away from the mean. Spellman et al data (C) were available in log-transformed format, and are depicted as a log2-fold change relative to mean. Each column (A-C) represents a time point in minutes. Despite drastically different culturing conditions between the three experiments, the temporal ordering and periodicity of gene expression is very similar across the three *S*. *cerevisiae* cell-cycle profiling experiments.
(TIF)

S5 Fig A core set of about 100 orthologous fungal genes is conserved in periodicity and in temporal expression between *Saccharomyces cerevisiae*, *Cryptococcus neoformans*, and *Candida albicans*. A list of 494 periodic genes in *C*. *albicans* was obtained from Cote et al 2009 [49]. Using FungiDB, the Candida Genome Database (CGD), and the original publication’s Supplemental Table 1, the *C*. *albicans* genes were mapped to 504 *S*. *cerevisiae* orthologs [46,49,81]. This *C*. *albicans*–*S*. *cerevisiae* list was crossed with the *S*. *cerevisiae*–*C*. *neoformans* orthologous, top periodic gene list from Fig 3. The final lists of *S*. *cerevisiae*–*C*. *neoformans*–*C*. *albicans* orthologs are shown here. The 96 unique *S*. *cerevisiae* genes are ordered on peak time expression, as in Fig 3 (A). The 89 unique *C*. *neoformans* genes (B) are ordered the same as their respective ortholog in A. Four replicates of microarray time series data from the *C*. *albicans* cell cycle were averaged together for the 92 unique probe IDs of interest, excluding missing data points, using R (C). In each heatmap, transcript levels are depicted as a z-score change relative to mean expression for each gene, where values represent the number of standard deviations away from the mean. Each row represents an orthologous periodic gene set, in the same order for (A-C) (for exact ordering of gene pairs and multiple-mappings, see S5 Table, Tab 2).
(TIF)

S6 Fig CLOCCS model fits and parameter estimates for aligning the time series data. The first, most synchronous cycle of budding data from *S*. *cerevisiae* and *C*. *neoformans* (Fig 1) was fed into the CLOCCS model [59,60]. The fraction of cells with a bud (filled circles) is shown for *S*. *cerevisiae* (blue) and *C*. *neoformans* (green) wild-type cells (A, reproduced from Fig 1). The CLOCCS predicted first-bud curves and associated uncertainty (purple band) is shown for *S*. *cerevisiae* and *C*. *neoformans*, respectively (C-D). The CLOCCS parameters are given in a table for each experiment, which contains the mean value and 95% confidence interval (in parentheses) for each model parameter (E). The mean values for cell-cycle period (λ) and recovery time (μ_0_) were used to align the two time series (Figs 4 and 6) by converting time points to scaled CLOCCS lifeline points (see S1 File). The scaled budding curves, aligned by CLOCCS lifeline points, are also shown here (B).
(TIF)

S7 Fig Quantification of peak times for budding, DNA replication, spindle assembly, and mitosis genes in *S*. *cerevisiae* and *C*. *neoformans* shown in Fig 4. Peak expression times for cell-cycle genes and orthologs in *S*. *cerevisiae* (blue) and *C*. *neoformans* (green) were found in the first cell cycle (see Fig 4A, 4B, 4D, 4E, 4G and 4H). Cycle 1 gene expression peak times were also found in the common cell-cycle timeline (CLOCCS lifeline point units) as described (see S1 File). Histograms of peak times for *S*. *cerevisiae* periodic budding genes (77) and orthologous *C*. *neoformans* genes (61) show peaks distributed throughout the first cell cycle (**A-B**). Histograms of peak times for *S*. *cerevisiae* periodic DNA replication genes (61) and orthologous *C*. *neoformans* genes (53) show a tight distribution of peak times in the mid-cell cycle and similar temporal ordering between the two yeasts (**C-D**). Histograms of peak times for *S*. *cerevisiae* periodic mitosis genes (143) and orthologous *C*. *neoformans* genes (87) show a similar range of peak times and that S-phase genes generally peak before M-phase genes in each yeast (**E-F**).
(TIF)

S8 Fig SBF/MBF binding site motifs are conserved in *C*. *neoformans* TF network orthologs and in periodic DNA replication genes. TF network genes (Table 1) were selected for conservation and edge connection to the putatively conserved G1/S motif (Fig 6; *CLB1*, *CLB2*, *CLB3*, *CLB4*, *CLB5*, *CLB6*, *CLN1*, *CLN2*, *FKH1*, *FKH2*, *HCM1*, *MBP1*, *SWI4*, *SWI6*, *WHI5*, *YHP1*, and *YOX1*) as well as their respective *C*. *neoformans* orthologs (CNAG_04575 (*CLB*s), CNAG_02095 (*CLB*s), CNAG_06092 (*CLN*s), CNAG_05861 (*FKH1*), CNAG_02566 (*FKH2*), CNAG_03116 (*HCM1*), CNAG_07464 (*MBP1*, *SWI4*), CNAG_01438 (SWI6), CNAG_05591 (WHI5), CNAG_05176 (YHP1), CNAG_04586 (YHP1), and CNAG_03229 (YOX1)). The promoter region for each gene was designated to be the 1000 base pairs upstream of the Start Codon, and all sequences were obtained from FungiDB [46]. The promoter sequences of 38 periodic DNA replication ortholog pairs were also obtained (ASF1/CNAG_00085, CDC45/CNAG_02406, CHL1/CNAG_04026, CLB6/CNAG_04575, CSM3/CNAG_04603, CTF4/CNAG_04662, DPB2/CNAG_06634, FKH1/CNAG_05861, FKH2/CNAG_02566, HHF1/CNAG_07807/CNAG_01648, HHT1/CNAG_04828/CNAG_06745, HTA2/CNAG_06747, HTB2/CNAG_06746, MCM6/CNAG_03962, MRC1/CNAG_03023, ORC1/CNAG_02195, POB3/CNAG_05661, POL1/CNAG_06607, POL12/CNAG_06142, POL2/CNAG_02654, POL3/CNAG_02563, POL30/CNAG_06079, PRI1/CNAG_02385, PRI2/CNAG_04742, PSF1/CNAG_03374, PSF3/CNAG_04682, RAD27/CNAG_00991, RFA1/CNAG_01144, RFA2/CNAG_01316, RFC1/CNAG_07539, RLF2/CNAG_01573, RNR1/CNAG_02208, RNR2/CNAG_01915, SPT16/CNAG_01726, TOF1/CNAG_07686, and TOP1/CNAG_04190). The promoter sequences of 36 *S*. *cerevisiae* genes and 38 *C*. *neoformans* genes were selected at random from the ordered gene lists (Fig 3A and 3C) as negative controls for periodic gene promoters (ALK2, APD1, BDF2, CLB1, CLB2, CLB6, COQ10, EMP24, FHL1, FZO1, GPA2, HOS3, IDP1, IMO32, KEL1, KIP3, KRE6, LIP5, LSC2, MSH6, NTO1, PHB2, PMC1, POL3, PRC1, PSR1, PYC1, PYC2, ROD1, SCP160, SEY1, SMC3, SMC4, VAC14, VBA1, YBR053C, CNAG_00320, CNAG_00498, CNAG_00770, CNAG_00811, CNAG_00991, CNAG_01055, CNAG_01167, CNAG_01372, CNAG_01461, CNAG_01566, CNAG_01622, CNAG_01750, CNAG_01844, CNAG_02022, CNAG_02154, CNAG_02229, CNAG_02447, CNAG_02506, CNAG_02793, CNAG_02927, CNAG_03395, CNAG_03453, CNAG_03743, CNAG_03845, CNAG_04033, CNAG_04168, CNAG_04170, CNAG_04900, CNAG_04961, CNAG_05003, CNAG_05144, CNAG_05934, CNAG_05977, CNAG_06087, CNAG_06665, CNAG_06819, CNAG_07322, CNAG_07635). The Weeder 2.0 program was used to identify enriched TF binding sites in the promoter regions for each gene list, using *S*. *cerevisiae* oligo frequency parameters (command line implementation:./weeder2 -f *path_to_promoters_fasta* -O SC -b 5 -maxm 25) [82]. The top-scoring enriched 10-mer motif is shown for 17 input *S*. *cerevisiae* TF network promoters (A), for 12 input *C*. *neoformans* TF network orthologs (B), for 36 input *S*. *cerevisiae* DNA replication promoters (C), for 38 input *C*. *neoformans* DNA replication promoters (D), for 36 input *S*. *cerevisiae* random periodic gene promoters (E), and for 38 input *C*. *neoformans* random periodic gene promoters (F). In each promoter list, the “CGCG” core of common nucleotides bound by MBF and SBF was identified in the top 6-, 8-, or 10-mer enriched motifs for 15/17 (A), 7/12 (B), 30/36 (C), and 35/38 (D) promoter sets, respectively [65]. The top-scoring 10-mer motif from each promoter set was visualized using the WebLogo interface and the enriched motif alignments output from Weeder 2.0 [83].
(TIF)
