# Supplementary material for: Investigating Conservation of the Cell-Cycle-Regulated Transcriptional Program in the Fungal Pathogen, Cryptococcus neoformans
Source: PLoS Genet. 2016 Dec 5;12(12):e1006453. doi: 10.1371/journal.pgen.1006453 (PMC5137879; doi:10.1371/journal.pgen.1006453)
Supplement: S1 File — This file contains further details on the RNA-Seq data analysis pipeline, periodic gene ranking algorithms, alignment of the two time series experiments using CLOCCS, and documentation of sequence orthologs. (DOCX) [file pgen.1006453.s001.docx]

**Supporting Information Methods to Accompany:**

“Investigating Conservation of the Cell-Cycle-Regulated Transcriptional Program in the Fungal Pathogen, *Cryptococcus neoformans*”

Christina M. Kelliher, Adam R. Leman, Crystal S. Sierra, and Steven B. Haase

**RNA-Sequencing data normalization**

*S. cerevisiae* total mRNA was prepared in libraries of stranded 50 base-pair single-end reads and multiplexed at 10 time point samples per sequencing lane. Illumina TruSeq Kit Stranded Library Preparation and HiSeq sequencing were performed at the Duke University Sequencing Facility*. C. neoformans* total mRNA was prepared in libraries of stranded 125 base-pair paired-end reads and multiplexed at 12 time point samples per sequencing lane. KAPA Kit Stranded Library Preparation and HiSeq sequencing were also performed at the Duke Sequencing Facility*.* Raw FASTQ files from each experiment were aligned to the respective yeast reference genome using STAR [1]. The *S. cerevisiae* S288C genome (Ensembl build R64-1-1) was downloaded from Illumina iGenomes on March 2, 2016 (https://support.illumina.com/sequencing/sequencing_software/igenome.html). The *C. neoformans* H99 genome was downloaded from the Broad Institute on March 2, 2016 (using “cryptococcus_neoformans_var._grubii_h99__cna3__3_supercontigs.fasta”) [2].

RNA-Seq mapping statistics are presented in the Table below. Reads mapping uniquely to annotated gene features were quantified using HTSeq-count [3]. For *S. cerevisiae*, the average total reads obtained from the 60 time point samples (multi-plexed across 6 sequencing lanes) was about 18.5M. The average reads mapping uniquely to the reference genome was about 16M (87%). The average reads mapping uniquely to an annotated gene in the reference genome was about 14.4M (78%). For *C. neoformans*, the average total reads obtained from the 24 time point samples (multi-plexed across 2 sequencing lanes) was about 24.1M. The average reads mapping uniquely to the reference genome was about 22.1M (92%). The average reads mapping uniquely to an annotated gene in the reference genome was about 21.1M (88%).

Transcript quantification of annotated yeast genes was performed using alignment files output from STAR and Cufflinks2 [4]. Time point samples from the respective yeasts were then normalized together using the CuffNorm feature. The normalized FPKM gene expression outputs (“genes.fpkm_table”) were used in the analyses presented. To avoid fractional and zero values, 1 was added to every FPKM value in each dataset using the R statistical programming environment [5]. Fractions and zeros were found to interfere with the periodicity algorithms that involved log-transformation of data points (data not shown). Normalized gene expression data for each yeast are available in S1 and S2 Tables. Raw RNA-Sequencing data from this manuscript have been submitted to the NCBI Gene Expression Omnibus (GEO; http://www.ncbi.nlm.nih.gov/geo/) under accession number GSE80474.

STAR, HTSeq, and Cufflinks default parameters were modified slightly to accommodate smaller, well-annotated yeast genomes (~12 Mb for *S. cerevisiae*, ~19 Mb for *C. neoformans* [6]). Below is a generalized summary of the UNIX/Linux analysis pipeline, with file paths and generic file names in *italics*.

Yeast RNA-Sequencing Data Pipeline:

1. Align reads:

STAR --runThreadN 1 --runMode alignReads --genomeDir *path_to_yeast_genome_build* --sjdbGTFfile *path_to_yeast_transcriptome_gtf* --readFilesIn *sample.fastq* --outFilterType BySJout --alignIntronMin 10 --alignIntronMax 3000 --outFileNamePrefix ./STAR_out/ --outFilterIntronMotifs RemoveNoncanonical

2. Count reads mapping to genes:

htseq-count --order=pos --stranded=reverse --mode=intersection-nonempty *sample.aligned.sorted.sam path_to_yeast_transcriptome_gtf* > *sample.txt*

3. Quantify transcripts:

cuffquant --library-type=fr-firststrand *path_to_yeast_transcriptome_gtf*

*sample.aligned.sorted.sam*

4. Normalize time series samples together:

cuffnorm --library-type=fr-firststrand *path_to_yeast_transcriptome_gtf* *.cxb

**Ranking wild-type periodic genes**

Time series gene expression data from *S. cerevisiae* and *C. neoformans* were run through four periodicity-ranking algorithms: persistent homology (PH), Lomb-Scargle (LS), JTK-CYCLE (JTK), and de Lichtenberg (DL) [7–11]. Each algorithm was implemented as described previously [12]. For both datasets, a period length of 75 minutes and range of 65-85 minutes were applied to search for and rank periodic genes.

In order to compare periodicity-ranking predictions, we chose to search the same range of periodic signals in the two yeast time series datasets. We justify this choice of 75-minute period length for both yeasts using CLOCCS algorithm fits to budding index data [13,14]. The first, most synchronous budding cycle from the *C. neoformans* time series yielded a predicted cell-cycle period (λ) of about 75 minutes (S6 Fig). The more synchronous three budding cycles from *S. cerevisiae* yielded a predicted cell-cycle period (λ) of about 69 minutes (Fig 1). As described previously [15], we also factored in the predicted daughter cell G1 delay (δ = 11 minutes) to the final estimate of 75 minutes for the *S. cerevisiae* cell-cycle period.

S1 and S2 Tables summarize the periodicity-ranking outputs and gene expression statistics for all *S. cerevisiae* and *C. neoformans* genes, respectively. We first examined the top 1600 periodic genes from each yeast (S1 Fig), as ~15-25% of the yeast genome has been labeled periodic by previous work. In order to validate our list of the top 1600 periodic genes in *S. cerevisiae*, we compared our list to 8 previously published cell-cycle periodic gene lists. *S. cerevisiae* standard gene name mappings between the 9 gene lists are described in the legend (S2 Fig). The periodic gene list presented in this study has the most genes in common with all other studies (75.9% average overlap), and the list from Bristow 2014 has the most unique genes (64.7% average overlap with 8 other studies) [15]. This result is encouraging because our large gene list has a large percent overlap with previous work. Interestingly, there are only 159 wild-type periodic genes in the intersection of all 9 studies. The cause of this fractional overlap is unknown. Our group and others have proposed that the following variables likely play a role in the generation of unique periodic gene sets: synchrony method, gene expression measurement platform, data normalization, and periodicity detection and/or algorithm score cutoff.

To further quantify the number of periodic genes in each yeast, we applied a quantitative score cutoff to the top 1600 ranked periodic genes. As described previously, we chose to filter on the Lomb-Scargle algorithm at a p-value cutoff of 0.5 [15], which yielded 1732 genes in *S. cerevisiae* and 1327 genes in *C. neoformans*. The LS algorithm was run a second time on a truncated version the *S. cerevisiae* dataset, where the time points exactly matched the sampling density of the *C. neoformans* experiment (i.e. 0-230 minutes, every 10 minutes). The magnitudes of p-values output from the LS algorithm are affected by time-series data sampling density, as described previously [12]. Therefore, to apply the same LS cutoff to each yeast dataset, we ran the same amount of data through the LS algorithm. We then took the intersection of genes in the top 1600 ranked by all algorithms and genes that passed the LS cutoff to identify 1246 periodic genes in *S. cerevisiae* and 1134 periodic genes in *C. neoformans* (Fig 2). Although we provide an estimate for the number of periodic genes in each yeast, we emphasize that there is a distribution of periodic shapes across all expressed genes (S1 Fig) [12]. Depending on the biological question at hand and quantitative cutoff used, different lists of genes labeled periodic will emerge (S2 Fig).

**Aligning budding yeast time series data**

In the previous section, we sought to identify and accurately compare top periodic gene sets between *S. cerevisiae* and *C. neoformans* (Fig 2-3). Here, we wanted to most accurately to align the two time series and compare the expression timing of sequence orthologs (Fig 4-6, gene expression line plots). To do this, we fit the first, most synchronous cycle of budding index data using the CLOCCS algorithm (S6 Fig). We then converted time points in minutes to scaled CLOCCS lifeline points using the estimated cell-cycle period (λ) and recovery time (μ_0_) parameters. Using *S. cerevisiae* data, the following example calculations apply to all time-to-lifeline conversions:

1. First budding cycle CLOCCS parameters:

λ = 78.44

μ_0_ = 23.99

2. Time-to-lifeline examples:

tp_1_ = 25 minutes

lp_1_ = (tp_1_ - μ_0_) / λ * 100 + 100 = 101.29 scaled lifeline points

tp_2_ = 30 minutes

lp_2_ = (tp_2_ – μ_0_) / λ * 100 + 100 = 107.66 scaled lifeline points

3. RecoveryTime-to-lifeline example:

lp_scale_ = lp_2_ – lp_1_ = 6.37 minutes

tp_1-recovery_ = 20 minutes

lp_1-recovery_ = lp_25min_ – lp_scale_ = 94.92 scaled lifeline points

**Documentation of sequence orthologs between *S. cerevisiae* and *C. neoformans***

To our knowledge, we have compiled the largest list to date of putative sequence orthologs between *C. neoformans* and *S. cerevisiae* from the literature, databases, and a few manual BLAST searches [6,16–18]. The results of the yeast ortholog compilation are presented in S4 Table.

4220 ortholog pairs were derived from the FungiDB database on July 17, 2015 by searching the 6962 *C. neoformans* annotated ORFs and performing the “Transform by Orthology” step to *S. cerevisiae* S288C genes. FungiDB utilizes the OrthoMCL algorithm to determine orthology between fungal genes [19]. The first steps of the OrthoMCL algorithm utilize “reciprocal BLAST criteria” to identify orthologs, where two orthologous sequences identify each other as the best match in both genomes of interest. OrthoMCL incorporates further steps to identify groups of paralogous and orthologous genes, but is built on the reciprocal BLAST criteria definition of orthologs.

848 ortholog pairs were derived from the publication of the *C. neoformans var. grubii* H99 genome [6]. In this study, Janbon and colleagues made significant improvements to the H99 transcriptome annotation at 1766 loci. They compared this new translated gene set (and the old predicted proteome) to *S. cerevisiae* protein sequences using BLAST, and hits with 30% identity or greater were reported in their Table S2. This method of ortholog identification utilizes “best hit criteria” only, which is not as strict of a definition as the reciprocal BLAST criteria implemented in OrthoMCL.

38 ortholog pairs were derived from a recent study of transcription factors in *C. neoformans* [18]. Supplementary Data 1 reported a list of transcription factors in *C. neoformans* and putative orthologs to *S. cerevisiae* derived from the DBD TF prediction database (http://www.transcriptionfactor.org/). The DBD TF prediction database utilizes the Pfam database to identify orthologous protein domains. The Pfam database is comprised of multiple sequence alignments of protein domain families from a variety of sequenced and annotated genomes. Protein sequences are then tested against protein family alignments, and orthologous domain hits are defined by HMMER algorithm parameters. Thus, this method of ortholog identification is similar to the “best hit criteria” definition for orthologous protein domains.

8 ortholog pairs were derived from BLAST searches using specific *S. cerevisiae* proteins of interest in the yeast cell-cycle network. BLASTp searches were performed using single *S. cerevisiae* FASTA sequence files (derived from FungiDB) against the Non-redundant protein sequences in *Cryptococcus neoformans var. grubii* H99 (taxid:235443). One exception, Whi5p (YOR083W), was searched using Pattern Hit Initiated BLAST (PHI-BLAST) and the GTB motif LXXRLXXAXXK (described previously in [20]) to identify its putative *C. neoformans* ortholog. Here, we are implementing the “best hit criteria” definition of orthologs, not the reciprocal BLAST criteria implemented in OrthoMCL.

The four above sources used a variety of definitions for orthology. Therefore, to compare the list of 4572 putative orthologs pairs in *S. cerevisiae* and *C. neoformans*, we performed a global alignment for all putative orthologous protein sequences in *S. cerevisiae* and *C. neoformans*. Global alignment scores were extracted for each pair of 4572 putative orthologs. This orthology test is similar to the “best hit criteria” definition, where two protein sequences must obtain a global E-value score less than or equal to 10 to be scored by the global alignment algorithm.

From the S4 Table, 3437 unique *S. cerevisiae* and 3405 unique *C. neoformans* protein sequences were obtained from FungiDB. We then used the global search feature in the FASTA program (http://fasta.bioch.virginia.edu/fasta/fasta_list.html) [21] to align all possible pairs of yeast proteins using the following command:

ggsearch36 cneo_3405_prot.fa scer_3437_prot.fa > output_all_global.txt

3593 / 4572 pairs of putative ortholog pairs showed a significant global alignment score (78.6%, E-value ≤ 10). 980 pairs of putative orthologs did not get scored in the global alignment (S4 Table, “NA” values). Each of the four sources contained predicted ortholog pairs that scored “NA” in our global alignment test (FungiDB: 712 / 4220 pairs, Janbon Table S2: 351 / 848 pairs, Jung Table S1: 18 / 38 pairs, Manual: 6 / 8 pairs). Each source used different criteria to determine orthology. The global alignment tool is optimal for evaluating orthologs with conservation throughout the protein sequence. Local alignment tools like BLAST and protein family identification tools would be best applied to further validate similar protein domains between proteins in these distantly related budding yeasts. We were inclusive in reporting evidence for all 4572 putative ortholog pairs in S4 Table for fungal biologists to reference and evaluate. All 4572 ortholog pairs described above were used to generate gene lists for Figs 3-6, Table 1, S5 Fig, S7 Fig, and S8 Fig.

*Saccharomyces cerevisiae* underwent a whole genome duplication (WGD) during evolution [22]. For the extant sets of *S. cerevisiae* WGD paralogs, the parsimonious expectation is that each paralog pair maps to one orthologous gene in *C. neoformans*. S4 Table lists *S. cerevisiae* genes that have a paralog from the WGD, which were derived from the Yeast Gene Order Browser (http://ygob.ucd.ie/) [23]. 915 / 4572 pairs of putative orthologs involve a *S. cerevisiae* gene with a WGD paralog. About 40% of these cases are parsimoniously explained, where both of the 2 *S. cerevisiae* paralogs map to 1 *C. neoformans* gene (348 / 915 cases). In a small fraction of pairs, only 1 of 2 *S. cerevisiae* paralogs was reported to have an ortholog in *C. neoformans* (110 / 915 cases). About half of the time, there were multiple mappings between *S. cerevisiae* paralogs and putative *C. neoformans* orthologs (457 / 915 cases). This phenomenon is likely due to large families of proteins with similar functions. For example, the family of sugar transporter genes in *S. cerevisiae* (e.g. *HXT1, HXT2, HXT3, HXT4, HXT5, HXT6, HXT7, HXT8, HXT9, HXT10, HXT11, HXT12, HXT13, HXT15, HXT16, HXT17*) maps to 8 putative *C. neoformans* orthologs (CNAG_03438, CNAG_03772, CNAG_04920, CNAG_04931, CNAG_05324, CNAG_05387, CNAG_06290, CNAG_06521).

The table of 4572 ortholog pairs was used to investigate conservation of cell-cycle genes in periodicity and in timing of expression (Fig 3-6, Table 1, S5, S7, and S8 Fig). S5-S7 Tables provide the exact gene lists of ortholog pairs used to generate Fig 3-5, respectively. In particular, S6 Table contains lists of known cell-cycle genes from *S. cerevisiae* from which we then identified *C. neoformans* orthologs [24–32].

**Supporting Information References**

1. Dobin A, Davis CA, Schlesinger F, Drenkow J, Zaleski C, Jha S, et al. STAR: Ultrafast universal RNA-seq aligner. Bioinformatics. 2013;29(1):15–21.

2. Cryptococcus neoformans var. grubii H99 Sequencing Project, Broad Institute of Harvard and MIT [Internet]. Available from: http://www.broadinstitute.org/

3. Anders S, Pyl PT, Huber W. HTSeq-A Python framework to work with high-throughput sequencing data. Bioinformatics. 2015;31(2):166–9.

4. Trapnell C, Hendrickson DG, Sauvageau M, Goff L, Rinn JL, Pachter L. Differential analysis of gene regulation at transcript resolution with RNA-seq. Nat Biotechnol. 2013;31(1):46–53.

5. R Core Development Team. R: a language and environment for statistical computing, 3.1.2 ed. [Internet]. R Foundation for Statistical Computing. 2014. Available from: https://www.r-project.org

6. Janbon G, Ormerod KL, Paulet D, Byrnes EJ, Yadav V, Chatterjee G, et al. Analysis of the Genome and Transcriptome of Cryptococcus neoformans var. grubii Reveals Complex RNA Expression and Microevolution Leading to Virulence Attenuation. PLoS Genet. 2014;10(4):e1004261.

7. Cohen-Steiner D, Edelsbrunner H, Harer J, Mileyko Y. Lipschitz Functions Have Lp-stable Persistence. Found Comput Math. 2010;10:127–39.

8. Lomb NR. Least-squares frequency analysis of unequally spaced data. Astrophys Space Sci. 1976;39(2):447–62.

9. Scargle JD. Studies in astronomical time series analysis. II - Statistical aspects of spectral analysis of unevenly spaced data. Astrophys J. 1982;263:835–53.

10. Hughes ME, Hogenesch JB, Kornacker K. JTK_CYCLE: an efficient nonparametric algorithm for detecting rhythmic components in genome-scale data sets. J Biol Rhythms. 2010;25(5):372–80.

11. de Lichtenberg U, Jensen LJ, Fausbøll A, Jensen TS, Bork P, Brunak S. Comparison of computational methods for the identification of cell cycle-regulated genes. Bioinformatics. 2005;21(7):1164–71.

12. Deckard A, Anafi RC, Hogenesch JB, Haase SB, Harer J. Design and analysis of large-scale biological rhythm studies: a comparison of algorithms for detecting periodic signals in biological data. Bioinformatics. 2013;29(24):3174–80.

13. Orlando DA, Lin CY, Bernard A, Iversen ES, Hartemink AJ, Haase SB. A probabilistic model for cell cycle distributions in synchrony experiments. Cell Cycle. 2007;6(4):478–88.

14. Orlando DA, Iversen ES, Hartemink AJ, Haase SB. A branching process model for flow cytometry and budding index measurements in cell synchrony experiments. Ann Appl Stat. 2009;3(4):1521–41.

15. Bristow SL, Leman AR, Simmons Kovacs LA, Deckard A, Harer J, Haase SB. Checkpoints couple transcription network oscillator dynamics to cell-cycle progression. Genome Biol. 2014;15(9):446.

16. Stajich JE, Harris T, Brunk BP, Brestelli J, Fischer S, Harb OS, et al. FungiDB: An integrated functional genomics database for fungi. Nucleic Acids Res. 2012;40(D1):675–81.

17. Johnson M, Zaretskaya I, Raytselis Y, Merezhuk Y, McGinnis S, Madden TL. NCBI BLAST: a better web interface. Nucleic Acids Res. 2008;36(Web Server issue):5–9.

18. Jung KW, Yang DH, Maeng S, Lee KT, So YS, Hong J, et al. Systematic functional profiling of transcription factor networks in Cryptococcus neoformans. Nat Commun. 2015;6:6757.

19. Li L, Stoeckert CJ, Roos DS. OrthoMCL: Identification of Ortholog Groups for Eukaryotic Genomes. Genome Res. 2003;13(9):2178–89.

20. Travesa A, Kalashnikova TI, de Bruin R a M, Cass SR, Chahwan C, Lee DE, et al. Repression of G1/S transcription is mediated via interaction of the GTB motifs of Nrm1 and Whi5 with Swi6. Mol Cell Biol. 2013;33(8):1476–86.

21. Pearson WR. Searching protein sequence libraries: Comparison of the sensitivity and selectivity of the Smith-Waterman and FASTA algorithms. Genomics. 1991;11(3):635–50.

22. Kellis M, Birren BW, Lander ES. Proof and evolutionary analysis of ancient genome duplication in the yeast Saccharomyces cerevisiae. Nature. 2004;428(6983):617–24.

23. Byrne KP, Wolfe KH. The Yeast Gene Order Browser: Combining curated homology and syntenic context reveals gene fate in polyploid species. Genome Res. 2005;15(10):1456–61.

24. Cherry JM, Hong EL, Amundsen C, Balakrishnan R, Binkley G, Chan ET, et al. Saccharomyces Genome Database: The genomics resource of budding yeast. Nucleic Acids Res. 2012;40(D1):700–5.

25. Bi E, Park HO. Cell polarization and cytokinesis in budding yeast. Genetics. 2012;191(2):347–87.

26. Howell AS, Lew DJ. Morphogenesis and the cell cycle. Genetics. 2012;190(1):51–77.

27. Masai H, Matsumoto S, You Z, Yoshizawa-Sugata N, Oda M. Eukaryotic Chromosome DNA Replication: Where, When, and How? Annu Rev Biochem. 2010;79(1):89–130.

28. Fragkos M, Ganier O, Coulombe P, Méchali M. DNA replication origin activation in space and time. Nat Rev Mol Cell Biol. 2015;16(6):360–74.

29. Weiss EL. Mitotic exit and separation of mother and daughter cells. Genetics. 2012;192(4):1165–202.

30. Biggins S. The composition, functions, and regulation of the budding yeast kinetochore. Genetics. 2013;194(4):817–46.

31. Winey M, Bloom K. Mitotic spindle form and function. Genetics. 2012;190(4):1197–224.

32. Marston AL. Chromosome segregation in budding yeast: Sister chromatid cohesion and related mechanisms. Genetics. 2014;196(1):31–63.
