# Supplementary figures and images for: Investigating Conservation of the Cell-Cycle-Regulated Transcriptional Program in the Fungal Pathogen, Cryptococcus neoformans
Source: PLoS Genet. 2016 Dec 5;12(12):e1006453. doi: 10.1371/journal.pgen.1006453 (PMC5137879; doi:10.1371/journal.pgen.1006453)

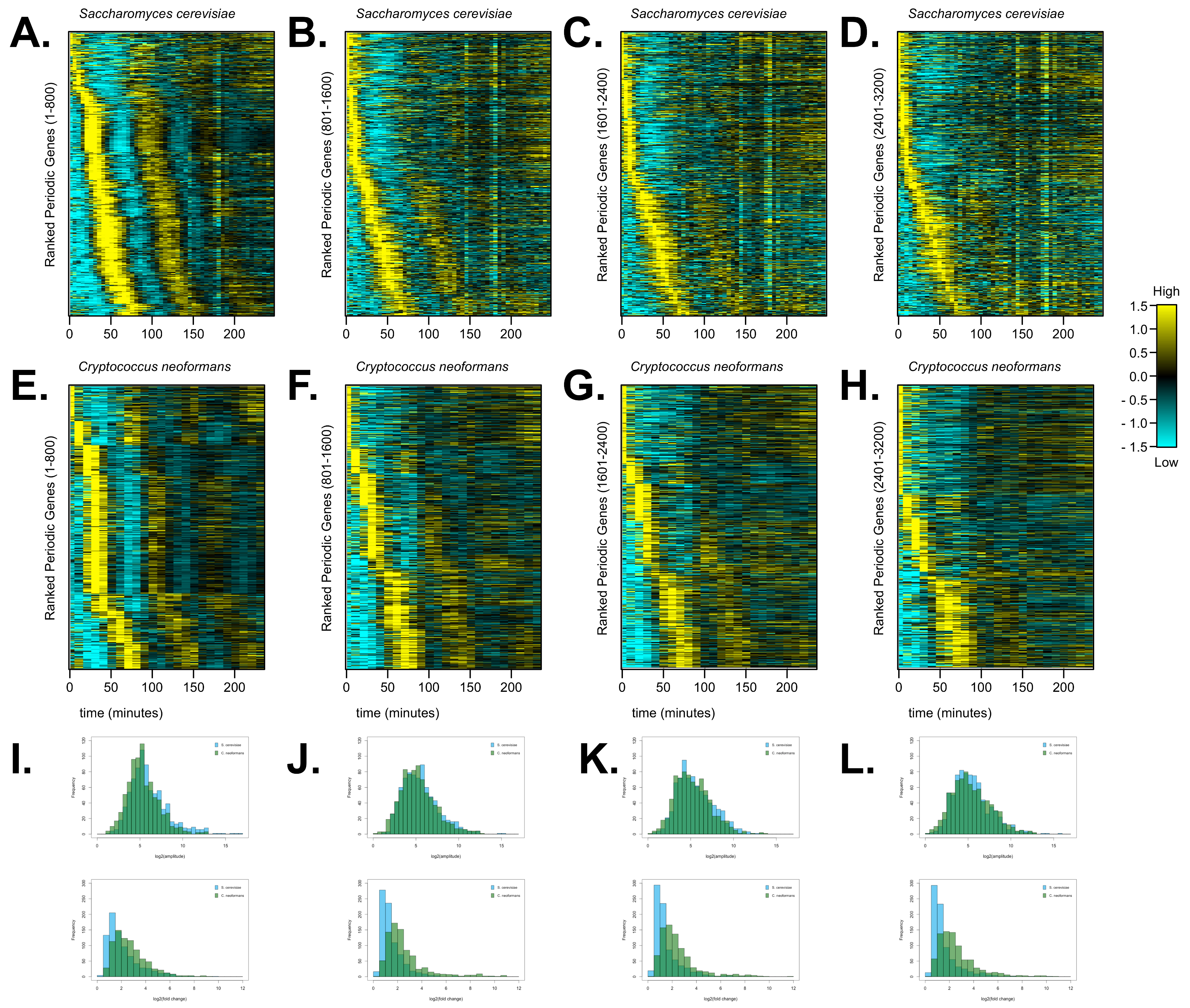

Supplement: S1 Fig — Four periodicity algorithms were run on both time series gene expression datasets at a period of 75 minutes. The top-ranked 1600 genes of S. cerevisiae (A-B) and C. neoformans (E-F) appear periodically expressed during the cell cycle. The next groups of ranked genes—1601–2400 (C, G) and 2401–3200 (D, H)—decay in periodic shape. However, there is no clear cutoff between “periodic” and “non-periodic” genes in either dataset. Transcript levels are depicted as a z-score change relative to mean expression for each gene. Each row represents a ranked periodic gene (see S1 and S2 Tables), and genes are ordered along the y-axis by peak expression during the cell cycle. Each column represents a time point in minutes. We also compared the distributions of amplitudes between S. cerevisiae (blue) and C. neoformans (green) ranked periodic genes (I-L). We examined two amplitude metrics—the absolute amplitude (max–min, top) and the fold-change amplitude (max / min, bottom). To compare the amplitude distributions, raw values were log2-normalized to make them normally distributed (I-L), and the following tests were conducted in R: wilcox.test, ks.test, var.test, and t.test. Distributions are statistically different for all fold-change histograms (I-L, bottom), where C. neoformans genes have higher mean fold-change values than S. cerevisiae genes. Distributions are statistically different for half of the absolute amplitude histograms (I, K, top), where S. cerevisiae genes have higher mean amplitude values than ranked C. neoformans genes. (TIF) [file pgen.1006453.s009.tif]

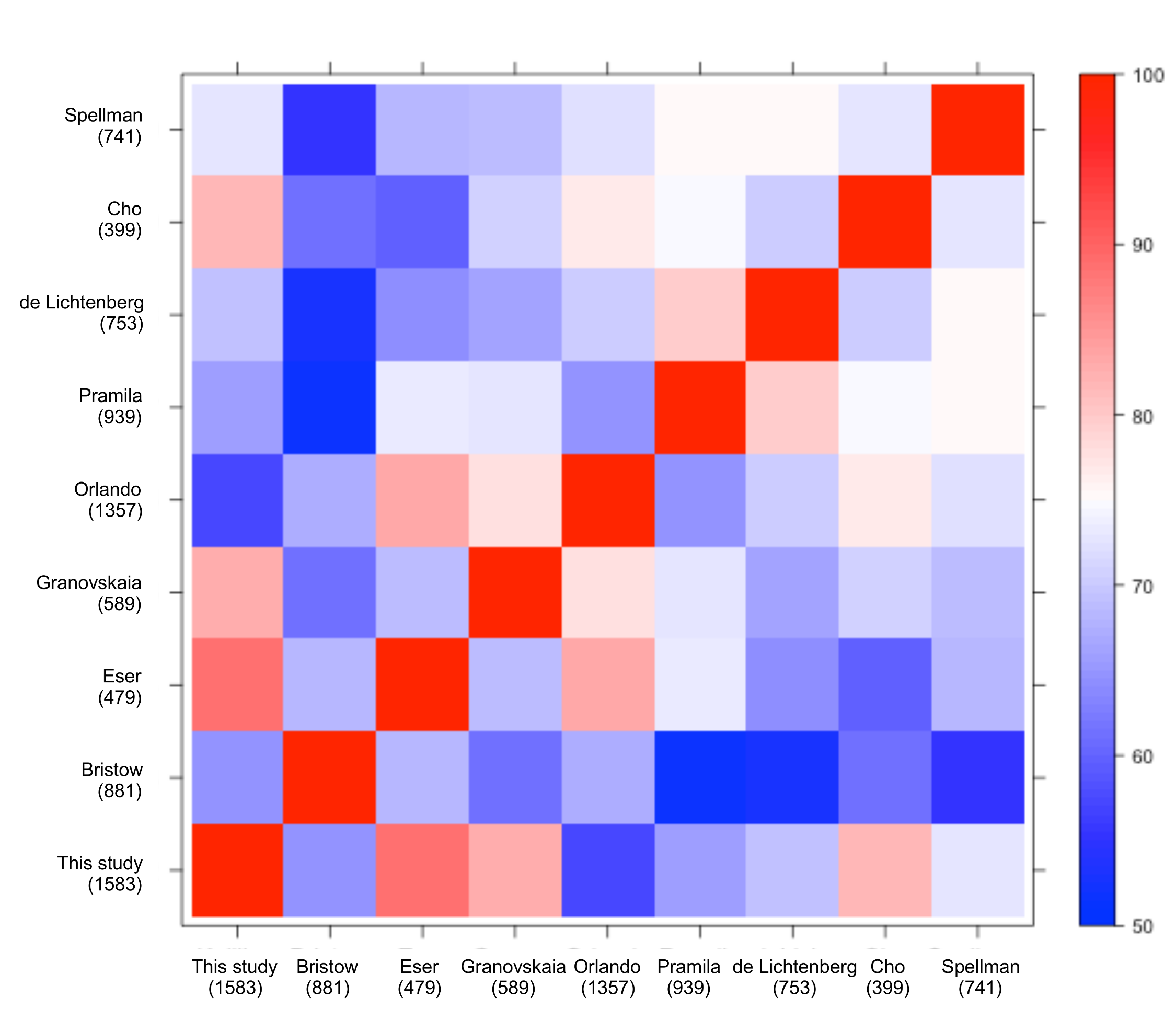

Supplement: S2 Fig — Periodic gene lists from each publication were derived as follows. The top 1600 genes from this study were converted to SGD standard names and 17 dubious ORFs were removed (1583 genes). The 856 microarray probe IDs from Bristow et al. Additional File 3 were converted to unique standard names (including duplicate probe ID mappings) to generate 881 genes (572 genes intersect with this study) [33]. The 479 genes from Eser et al. Addendum Table S6 were converted to standard names (425 intersect this study) [45]. The 598 genes from Granovskaia et al. Supplement Table 5 were converted to standard names, and 9 dubious ORFs were removed to generate 589 genes (487 intersect this study) [44]. The 1275 probe IDs from Orlando et al. Supplement Table 1 were converted to unique standard names to generate 1357 genes (777 intersect this study) [15]. The 991 genes from Pramila et al. with PBM5 rankings of 1000 or less were taken from Orlando et al., and 52 dubious ORFs were removed to generate 939 genes (618 intersect this study) [14]. The top 800 genes were taken from de Lichtenberg et al. (http://www.cbs.dtu.dk/cellcycle/yeast_benchmark/benchmark.php), and 47 dubious ORFs were removed to generate 753 genes (522 intersect this study) [41]. The 421 genes from Cho et al. were also taken from the de Lichtenberg et al. webpage, and 22 dubious ORFs were removed to generate 399 genes (326 intersect this study) [13]. The 800 genes from Spellman et al. were taken directly from the Supplement (http://genome-www.stanford.edu/cellcycle/data/rawdata/CellCycle95.xls), and 59 dubious ORFs were removed to generate 741 genes (540 intersect this study) [12]. Percent overlaps between each periodic gene list were calculated by dividing the number of intersecting genes by the total number of genes in the smaller list. Percent overlap is presented as a heatmap, and gene lists are ordered by date of publication. (TIF) [file pgen.1006453.s010.tif]

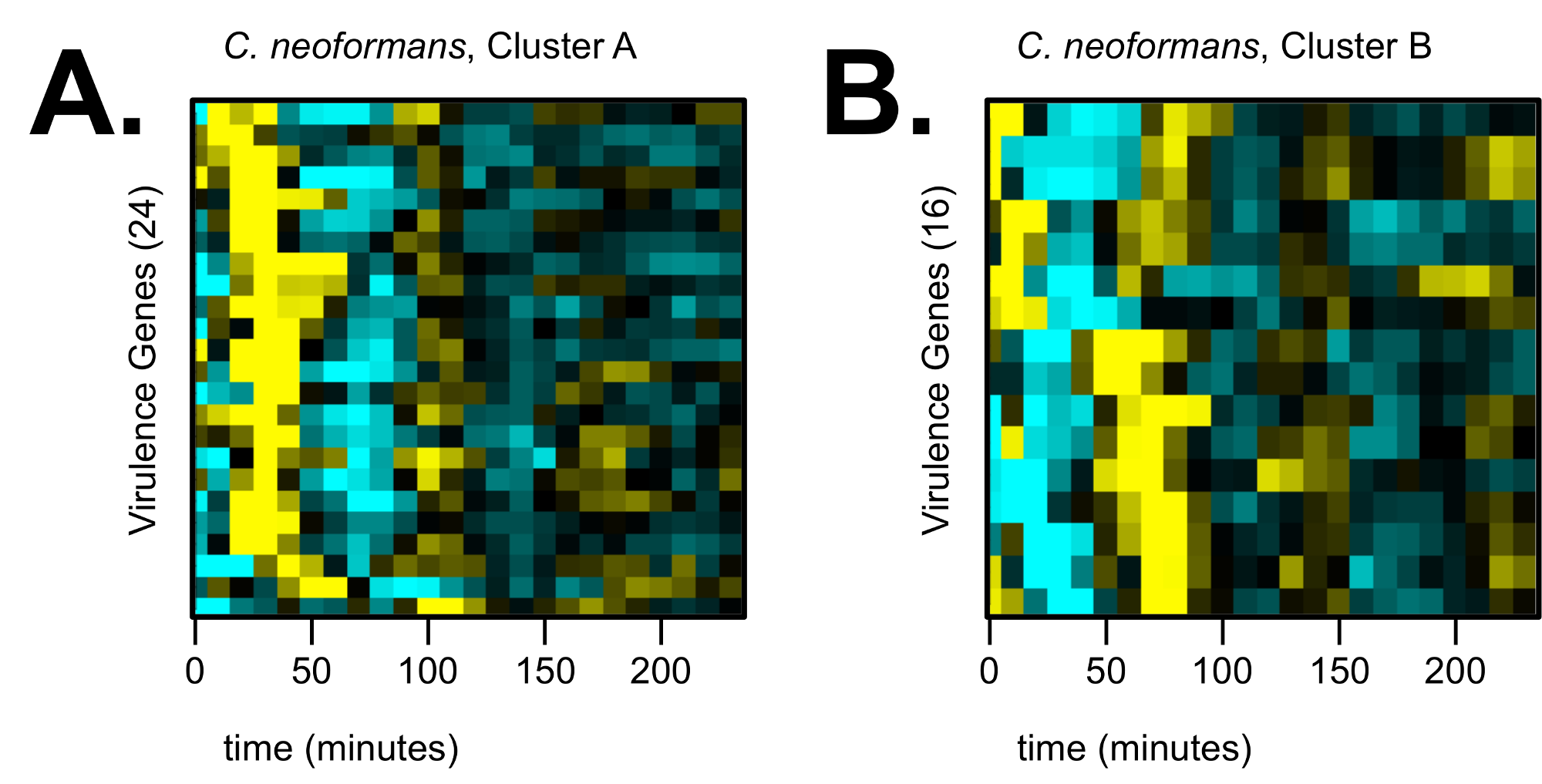

Supplement: S3 Fig — 40 periodic genes associated with virulence phenotypes from previous work (S3 Table) were clustered by an affinity propagation algorithm, as described in [15]. The 24 genes in Cluster A peak in an early-to-mid cell-cycle phase. The 16 genes in Cluster B are expressed approximately anti-phase to the Cluster A periodic genes. 11/14 periodic virulence genes associated with capsule and cell wall synthesis in C. neoformans belong to Cluster A (see S3 Table). (TIF) [file pgen.1006453.s011.tif]

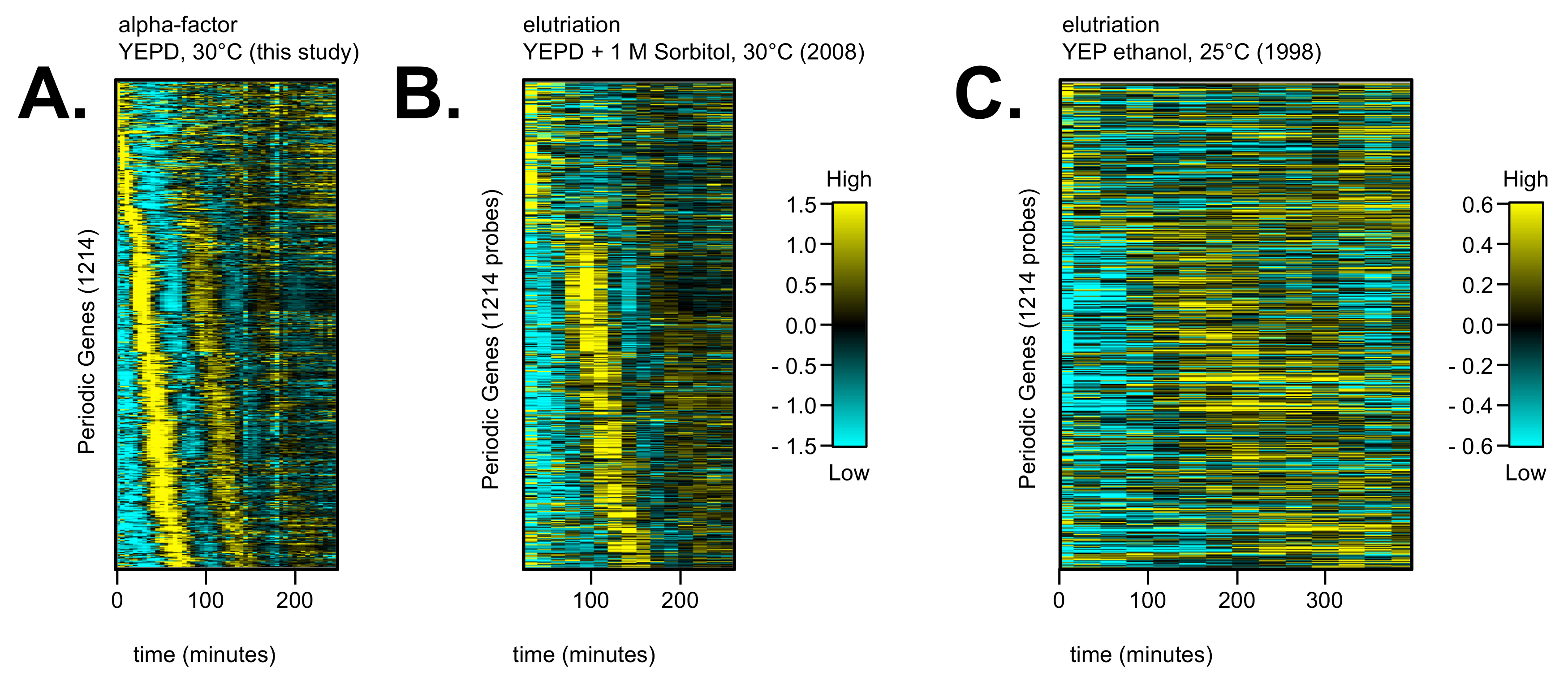

Supplement: S4 Fig — Microarray data was obtained from two different studies that profiled gene expression dynamics from wild-type yeast upon release from elutriation synchrony: Spellman 1998 [12] and Orlando 2008 [15]. Spellman and colleagues cultured the lab strain DBY7286 in YEP 2% ethanol at 25°C, elutriated, and released early G1 cells at 25°C. Orlando and colleagues cultured the lab strain 15D in YEP 2% galactose at 30°C, elutriated, and released early G1 cells into YEP 2% dextrose + 1 M Sorbitol at 30°C. In this study, cells were cultured in YEP 2% dextrose, arrested using alpha-factor, and G1 cells were released into YEP 2% dextrose at 30°C. 1214 out of 1246 periodic genes from this study (Fig 2A) were successfully mapped back to microarray probe IDs from the Affymetrix Yeast 2.0 array (Orlando) and to spots on custom-printed Cy3-Cy5 arrays (Spellman). In each heatmap, the 1214 genes were ordered in the exact same order along the y-axis by peak time of expression in the dataset from this study. For this study (A) and Orlando et al data (B), transcript levels are depicted as a z-score change relative to mean expression for each gene, where values represent the number of standard deviations away from the mean. Spellman et al data (C) were available in log-transformed format, and are depicted as a log2-fold change relative to mean. Each column (A-C) represents a time point in minutes. Despite drastically different culturing conditions between the three experiments, the temporal ordering and periodicity of gene expression is very similar across the three S. cerevisiae cell-cycle profiling experiments. (TIF) [file pgen.1006453.s012.tif]

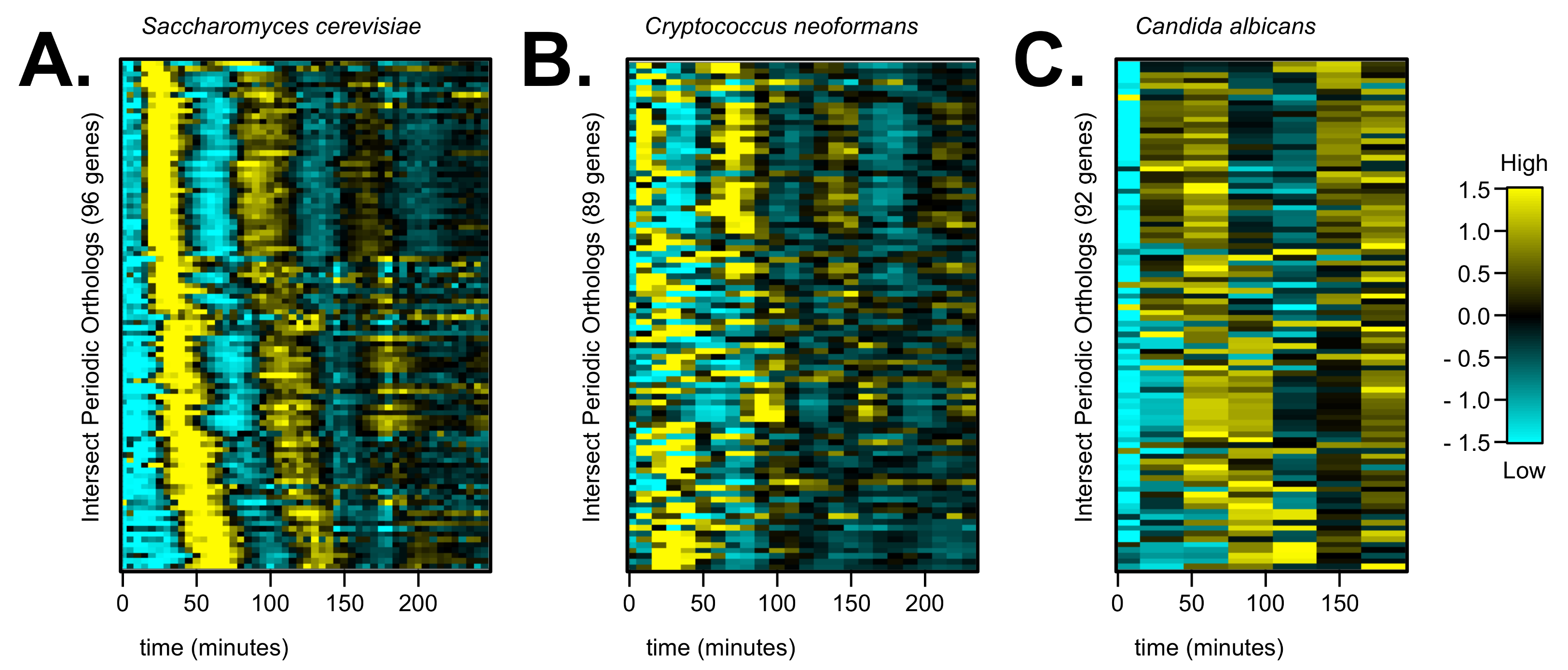

Supplement: S5 Fig — A list of 494 periodic genes in C. albicans was obtained from Cote et al 2009 [49]. Using FungiDB, the Candida Genome Database (CGD), and the original publication’s Supplemental Table 1, the C. albicans genes were mapped to 504 S. cerevisiae orthologs [46,49,81]. This C. albicans–S. cerevisiae list was crossed with the S. cerevisiae–C. neoformans orthologous, top periodic gene list from Fig 3. The final lists of S. cerevisiae–C. neoformans–C. albicans orthologs are shown here. The 96 unique S. cerevisiae genes are ordered on peak time expression, as in Fig 3 (A). The 89 unique C. neoformans genes (B) are ordered the same as their respective ortholog in A. Four replicates of microarray time series data from the C. albicans cell cycle were averaged together for the 92 unique probe IDs of interest, excluding missing data points, using R (C). In each heatmap, transcript levels are depicted as a z-score change relative to mean expression for each gene, where values represent the number of standard deviations away from the mean. Each row represents an orthologous periodic gene set, in the same order for (A-C) (for exact ordering of gene pairs and multiple-mappings, see S5 Table, Tab 2). (TIF) [file pgen.1006453.s013.tif]

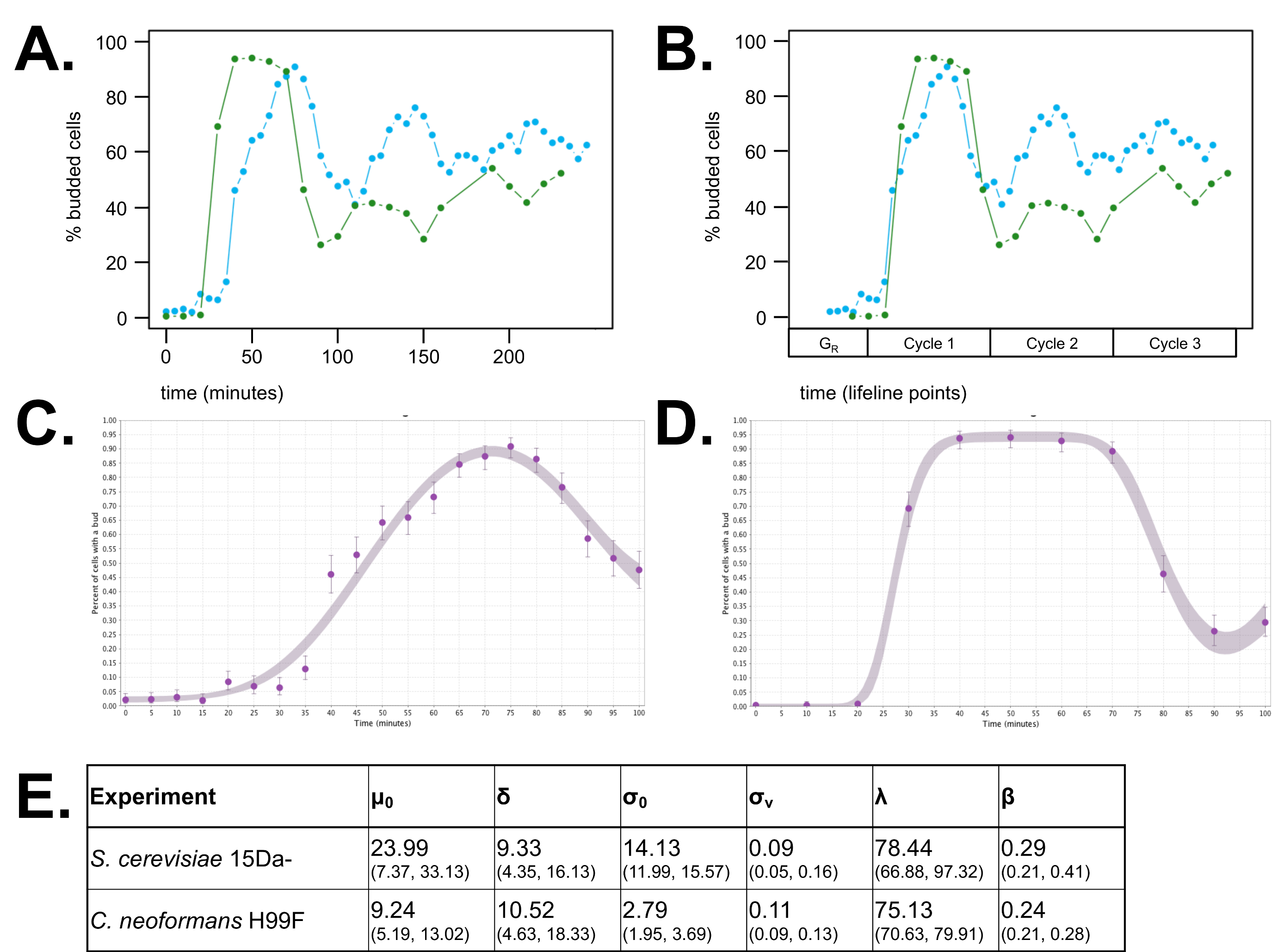

Supplement: S6 Fig — The first, most synchronous cycle of budding data from S. cerevisiae and C. neoformans (Fig 1) was fed into the CLOCCS model [59,60]. The fraction of cells with a bud (filled circles) is shown for S. cerevisiae (blue) and C. neoformans (green) wild-type cells (A, reproduced from Fig 1). The CLOCCS predicted first-bud curves and associated uncertainty (purple band) is shown for S. cerevisiae and C. neoformans, respectively (C-D). The CLOCCS parameters are given in a table for each experiment, which contains the mean value and 95% confidence interval (in parentheses) for each model parameter (E). The mean values for cell-cycle period (λ) and recovery time (μ0) were used to align the two time series (Figs 4 and 6) by converting time points to scaled CLOCCS lifeline points (see S1 File). The scaled budding curves, aligned by CLOCCS lifeline points, are also shown here (B). (TIF) [file pgen.1006453.s014.tif]

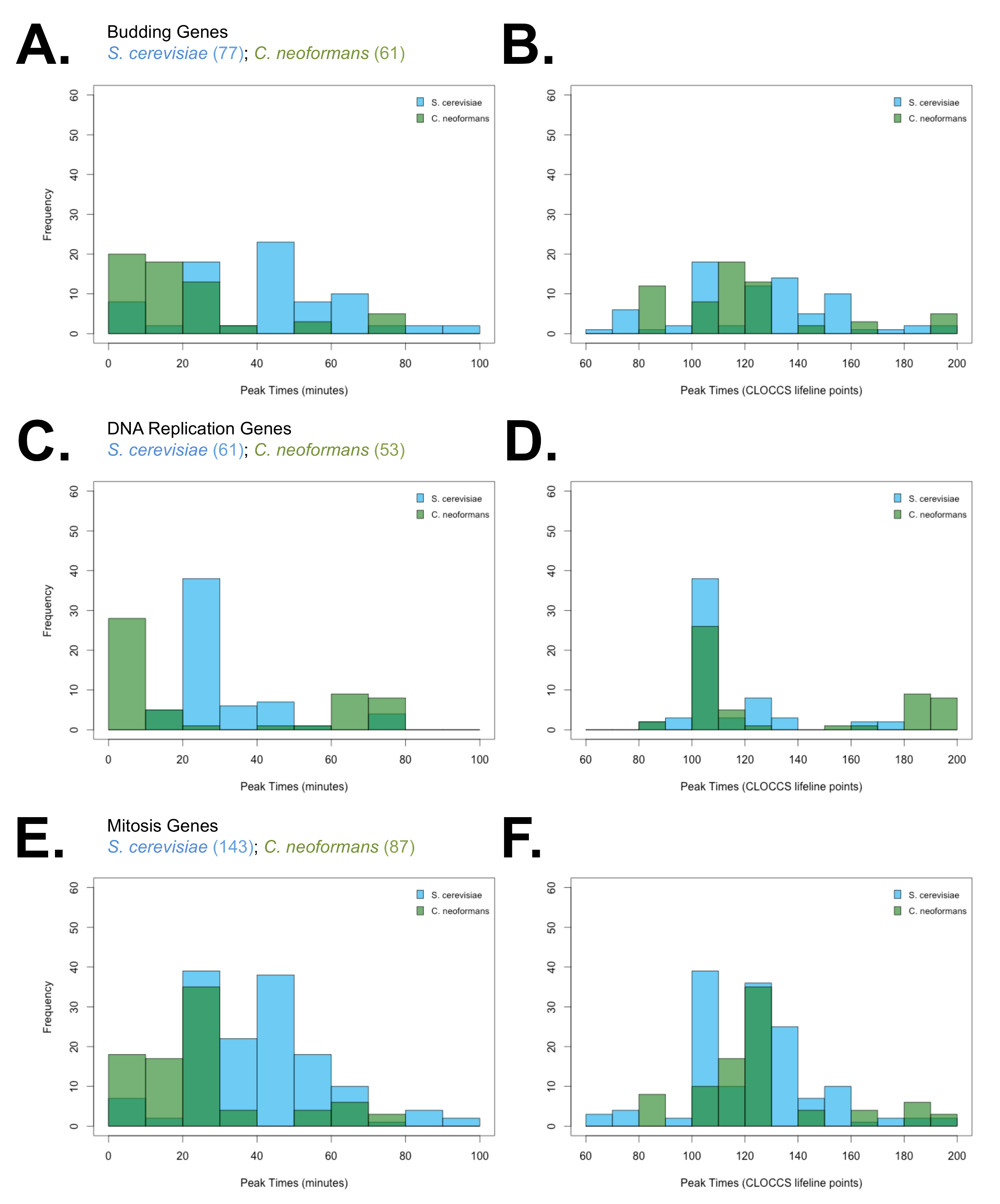

Supplement: S7 Fig — Peak expression times for cell-cycle genes and orthologs in S. cerevisiae (blue) and C. neoformans (green) were found in the first cell cycle (see Fig 4A, 4B, 4D, 4E, 4G and 4H). Cycle 1 gene expression peak times were also found in the common cell-cycle timeline (CLOCCS lifeline point units) as described (see S1 File). Histograms of peak times for S. cerevisiae periodic budding genes (77) and orthologous C. neoformans genes (61) show peaks distributed throughout the first cell cycle (A-B). Histograms of peak times for S. cerevisiae periodic DNA replication genes (61) and orthologous C. neoformans genes (53) show a tight distribution of peak times in the mid-cell cycle and similar temporal ordering between the two yeasts (C-D). Histograms of peak times for S. cerevisiae periodic mitosis genes (143) and orthologous C. neoformans genes (87) show a similar range of peak times and that S-phase genes generally peak before M-phase genes in each yeast (E-F). (TIF) [file pgen.1006453.s015.tif]

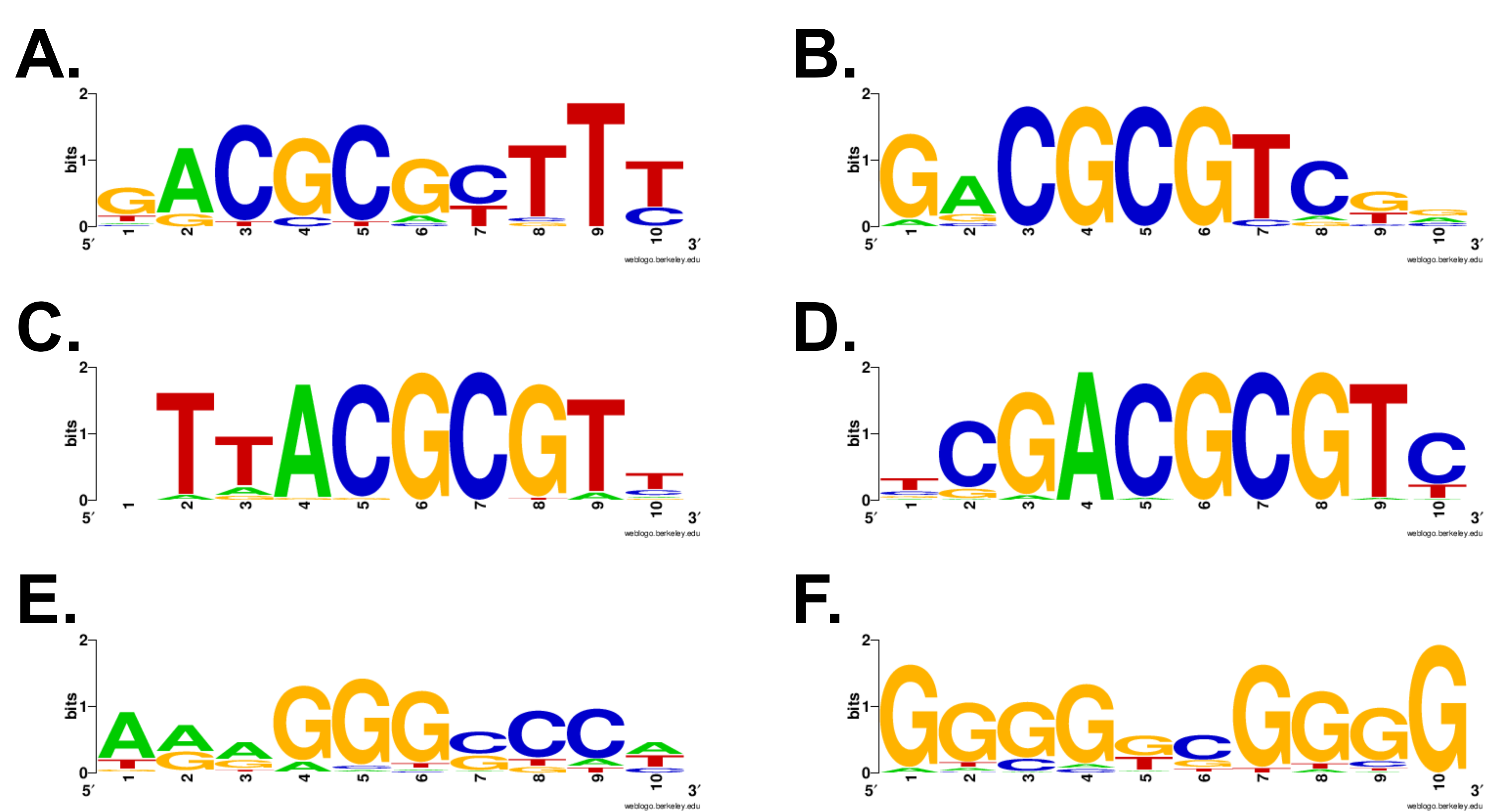

Supplement: S8 Fig — TF network genes (Table 1) were selected for conservation and edge connection to the putatively conserved G1/S motif (Fig 6; CLB1, CLB2, CLB3, CLB4, CLB5, CLB6, CLN1, CLN2, FKH1, FKH2, HCM1, MBP1, SWI4, SWI6, WHI5, YHP1, and YOX1) as well as their respective C. neoformans orthologs (CNAG_04575 (CLBs), CNAG_02095 (CLBs), CNAG_06092 (CLNs), CNAG_05861 (FKH1), CNAG_02566 (FKH2), CNAG_03116 (HCM1), CNAG_07464 (MBP1, SWI4), CNAG_01438 (SWI6), CNAG_05591 (WHI5), CNAG_05176 (YHP1), CNAG_04586 (YHP1), and CNAG_03229 (YOX1)). The promoter region for each gene was designated to be the 1000 base pairs upstream of the Start Codon, and all sequences were obtained from FungiDB [46]. The promoter sequences of 38 periodic DNA replication ortholog pairs were also obtained (ASF1/CNAG_00085, CDC45/CNAG_02406, CHL1/CNAG_04026, CLB6/CNAG_04575, CSM3/CNAG_04603, CTF4/CNAG_04662, DPB2/CNAG_06634, FKH1/CNAG_05861, FKH2/CNAG_02566, HHF1/CNAG_07807/CNAG_01648, HHT1/CNAG_04828/CNAG_06745, HTA2/CNAG_06747, HTB2/CNAG_06746, MCM6/CNAG_03962, MRC1/CNAG_03023, ORC1/CNAG_02195, POB3/CNAG_05661, POL1/CNAG_06607, POL12/CNAG_06142, POL2/CNAG_02654, POL3/CNAG_02563, POL30/CNAG_06079, PRI1/CNAG_02385, PRI2/CNAG_04742, PSF1/CNAG_03374, PSF3/CNAG_04682, RAD27/CNAG_00991, RFA1/CNAG_01144, RFA2/CNAG_01316, RFC1/CNAG_07539, RLF2/CNAG_01573, RNR1/CNAG_02208, RNR2/CNAG_01915, SPT16/CNAG_01726, TOF1/CNAG_07686, and TOP1/CNAG_04190). The promoter sequences of 36 S. cerevisiae genes and 38 C. neoformans genes were selected at random from the ordered gene lists (Fig 3A and 3C) as negative controls for periodic gene promoters (ALK2, APD1, BDF2, CLB1, CLB2, CLB6, COQ10, EMP24, FHL1, FZO1, GPA2, HOS3, IDP1, IMO32, KEL1, KIP3, KRE6, LIP5, LSC2, MSH6, NTO1, PHB2, PMC1, POL3, PRC1, PSR1, PYC1, PYC2, ROD1, SCP160, SEY1, SMC3, SMC4, VAC14, VBA1, YBR053C, CNAG_00320, CNAG_00498, CNAG_00770, CNAG_00811, CNAG_00991, CNAG_01055, CNAG_01167, CNAG_01372, CNAG_01461, CNAG_01566, CNAG_01622, CNAG_01750, CNAG_01844, CNAG_02022, CN [file pgen.1006453.s016.tif]
